# Case study of the convergent evolution in the color patterns in the freshwater bivalves

**DOI:** 10.1038/s41598-022-14469-3

**Published:** 2022-07-13

**Authors:** Kaito Asato, Kentaro Nakayama, Takuya Imai

**Affiliations:** 1 Fukui Prefectural Dinosaur Museum, 51-11 Terao, Muroko, Katsuyama, Fukui 911-8601 Japan; 2 Institute of Dinosaur Research, Fukui Prefectural University, Katsuyama, Japan

**Keywords:** Palaeoecology, Evolutionary ecology, Palaeontology, Taxonomy, Evolution, Ecology

## Abstract

The class Bivalvia (phylum Mollusca) is one of the most successful at survival groups of animals with diverse color patterns on their shells, and they are occasionally preserved in the fossil record as residual color patterns. However, the fossil record of the residual color patterns in freshwater bivalves could be traced only to the Miocene, greatly limiting color pattern evolution knowledge. We present the color patterns of the Cretaceous freshwater bivalves belonging to three extinct families of the order Trigoniida (hereinafter the Kitadani Freshwater Bivalves) from Japan, which is the oldest and the second fossil record of freshwater molluscan color patterns. The Kitadani Freshwater Bivalves consists of two types of color patterns: stripes along the growth lines and radial rays tapered toward the umbo, which resemble that of the colored bands of extant freshwater bivalves. This resemblance of the color patterns between the Kitadani Freshwater Bivalves and the extant species indicates that the color patterns of the freshwater bivalves represent the convergent evolution between Trigoniida and Unionida. To explain this convergent evolution, we advocate three conceivable factors: the phylogenetic constraints, monotonous habitats typical of freshwater ecosystems, and the predation pressure by visual predators in freshwater sediments.

## Introduction

The class Bivalvia (phylum Mollusca) is one of the most successful at survival groups of animals with diverse color patterns on their shells^1^, particularly in marine bivalves^2^. The color pattern diversity of marine taxa has been attributed to their adaptation to their varying habitats^3–7^, while the phylogenic constraints for diversity are considered minor^8^. The color patterns are occasionally preserved even in the fossil record as residual color patterns^3,9,10^, with Devonian^11^ being the oldest.

In contrast to marine species, extant freshwater bivalves are monotonous and less diverse in their shell color^8^ and patterns^12–15^. Hence, whether the diversity of their color patterns remained low from the beginning or decreased through their evolutionary history greatly interests evolutionary biologists. The fossil record of the residual color patterns in freshwater bivalves could be traced only to the Miocene (about 15 Ma)^16^, severely limiting our knowledge about the evolution of their color patterns.

We explore the color patterns of freshwater bivalves from the Early Cretaceous Kitadani Formation, Fukui, central Japan, allowing us to trace their fossil record back to approximately 120 million years. Freshwater bivalves from the Kitadani Formation exhibit residual color patterns similar to some extant taxa, demonstrating the convergent evolution considering the color patterns, and suggesting that the color pattern similarity has remained for at least 120 million years.

## Results

### Residual color patterns preserved in the Cretaceous freshwater bivalves

**Figure 1:**
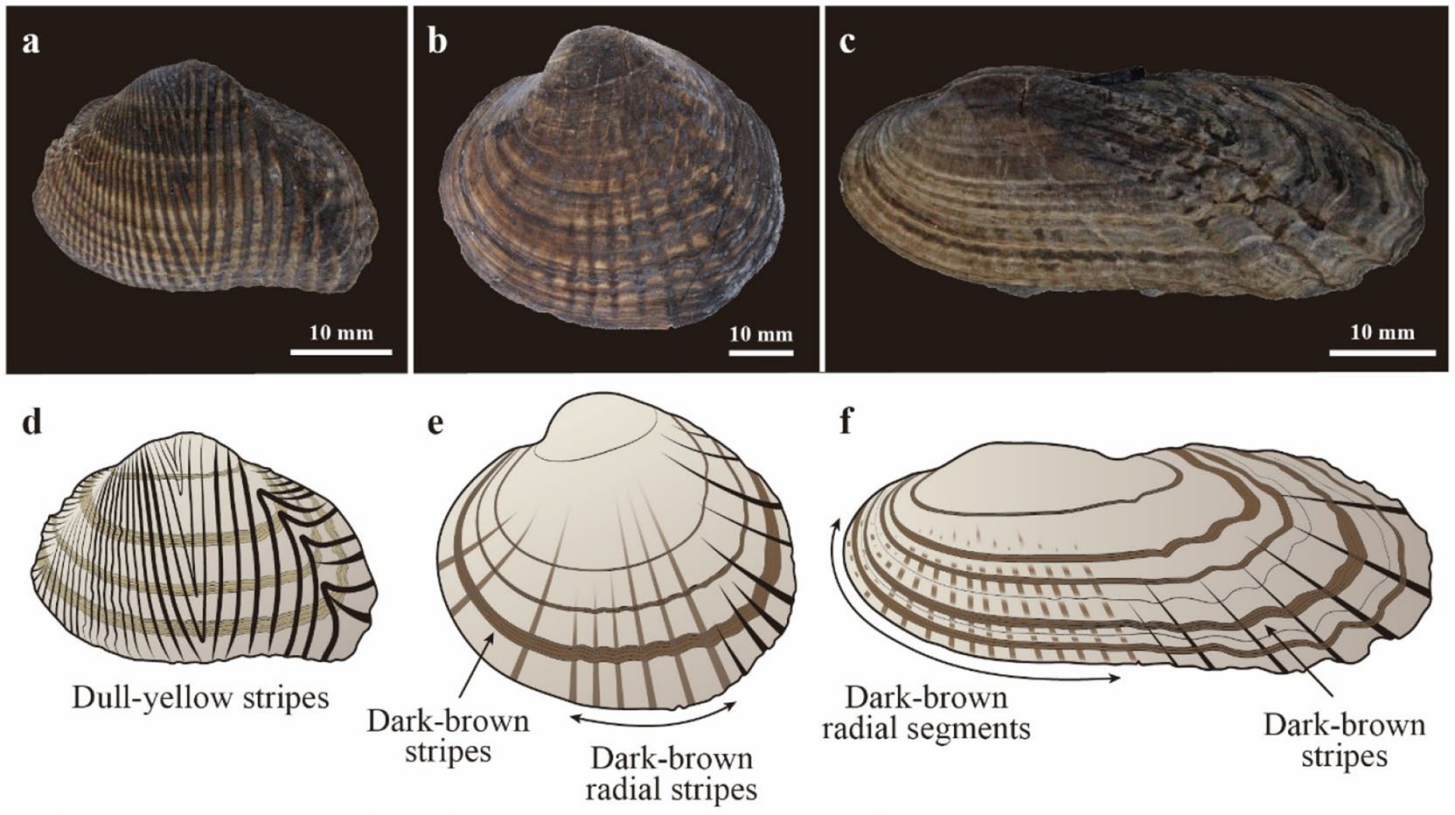
Color patterns of the Kitadani Freshwater Bivalves. (**a**–**c**) Water-immersion photographs of the Kitadani Freshwater Bivalves showing residual color patterns. (**a**) *T*. *tetoriensis*, FPDM-I-3445; (**b**) *M*. *matsumotoi*, FPDM-I-3456; (**c**) *P*. *naktongensis*, FPDM-I-3451. (**d**–**f**) schematic diagrams of reconstructed color patterns of the Kitadani Freshwater Bivalves. (**d**) *T*. *tetoriensis*; (**e**) *M*. *matsumotoi*; (**f**) *P*. *naktongensis*. Each color pattern was drawn based on the following specimens: (**d**) Fig. 1a, Extended Data Figs. 8a,c, 9a,c and 10a,c. (**e**) Fig. 1b, Extended Data Figs. 17a,c, 18a,c and 19a,c. (**c**) Fig. 1c, Extended Data Figs. 12a,c, 13a,c, 14a,c and 15a.

Abundant fossil molluscan remains in the Kitadani Formation (Aptian, about 120 Ma) of the Tetori Group cropping out in the Kitadani Dinosaur Quarry, Katsuyama, Fukui, Japan^17^. The Kitadani Formation represents fluvial, lacustrine, and floodplain deposits, yielding various terrestrial to freshwater fauna and flora^18^. Among these remains, three species of dominant freshwater bivalves belonging to the order Trigoniida bear dark-colored stripes on their shells, i.e., †*Matsumotoina matsumotoi* (S.-Y. Yang, 1979) [†Pseudohyriidae]; †*Plicatounio naktongensis* Kobayashi & Suzuki, 1936 [†Plicatounionidae]; and †*Trigonioides tetoriensis* Maeda, 1963 [†Trigonioididae] (hereinafter the Kitadani Freshwater Bivalves) (Fig. 1a–c)^19^. †*Matsumotoina*. *matsumotoi*, characterized by trigonally suboval shells with radial ribs and growth lines (Extended Data Figs. 16, 17b,d, 18b,d, 19b,d, see Supplementary Information S1), exhibits 1–3 mm wide bands distributed along the growth lines with 3–5 mm intervals, and the other form 2–3 mm wide bands radiating toward the ventral side with 3–5 mm intervals (Fig. 1b,e, Extended Data Figs. 17a,c, 18a,c, 19a,c). The radial stripes reside on the interspace of the plicated ribs on two-thirds of the posteroventral side of the shells and extend from the ventral shell margin to the median shell. In †*P*. *naktongensis* having elongated elliptical shells with radial plicated ribs and growth lines (Extended Data Figs. 11, 12b,d, 13b,d, 14b,d, 15b, see Supplementary Information S1), there are more bands running along the growth lines than those in †*M*. *matsumotoi* and sinuous near the posterior plicated ribs (Fig. 1c,f, Extended Data Figs. 12a,c, 13a,c, 14a,c, 15a). Additionally, †*P*. *naktongensis* bears colored axial segments that are 2–3 mm wide and arranged radially on the anteroventral portion. In †*T*. *tetoriensis* featured by subtrigonal shells with V-shaped ribs (Extended Data Figs. 7, 8b,d, 9b,d, 10b,d, see Supplementary Information S1), five to seven 1–5 mm wide dark stripes, appear along the growth lines, whereas radial stripes are absent, unlike †*M*. *matsumotoi* and †*P*. *naktongensis* (Fig. 1a,d, Extended Data Figs. 8a,c, 9a,c, 10 a,c).

### Color patterns of extant freshwater bivalves

**Figure 2:**
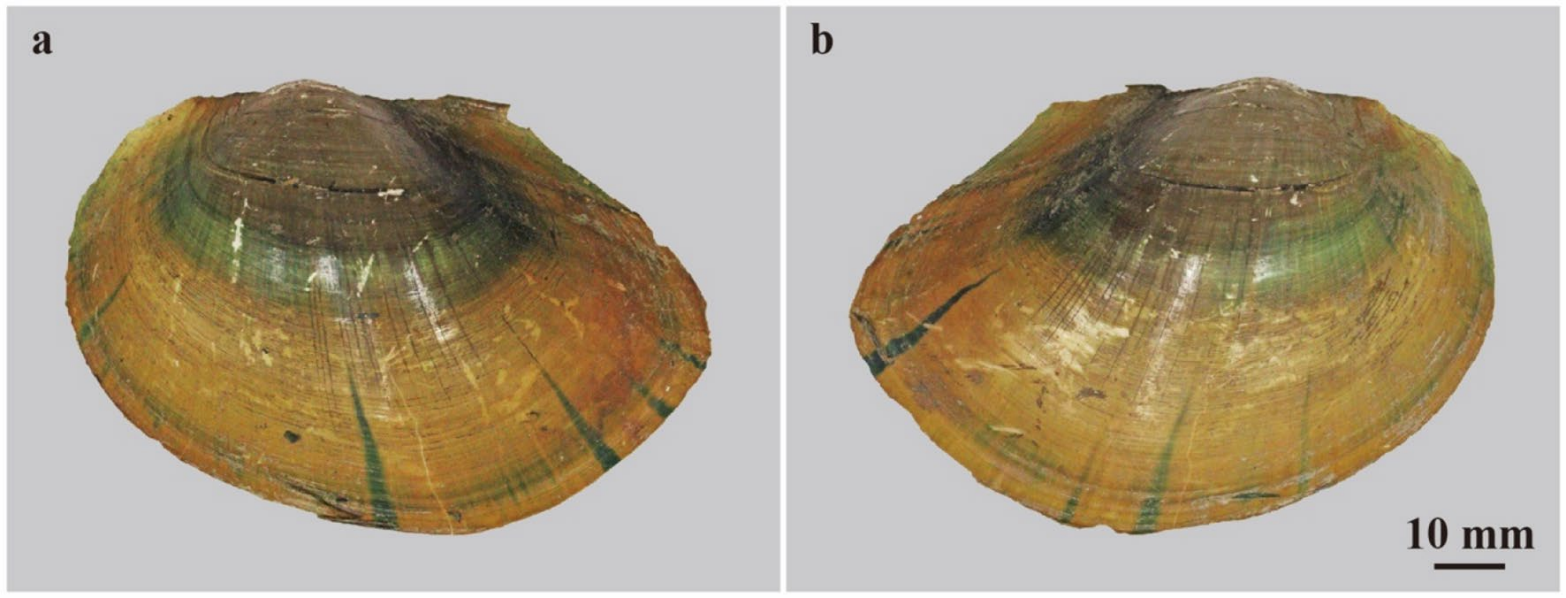
Color patterns of the extant freshwater bivalve *Sinanodonta calipygos*. (**a,b**) FPDM-I-0002924 from Lake Biwa, Shiga, Central Japan. (**a**) Left valve under normal light; (**b**) right valve under normal light.

**Figure 3:**
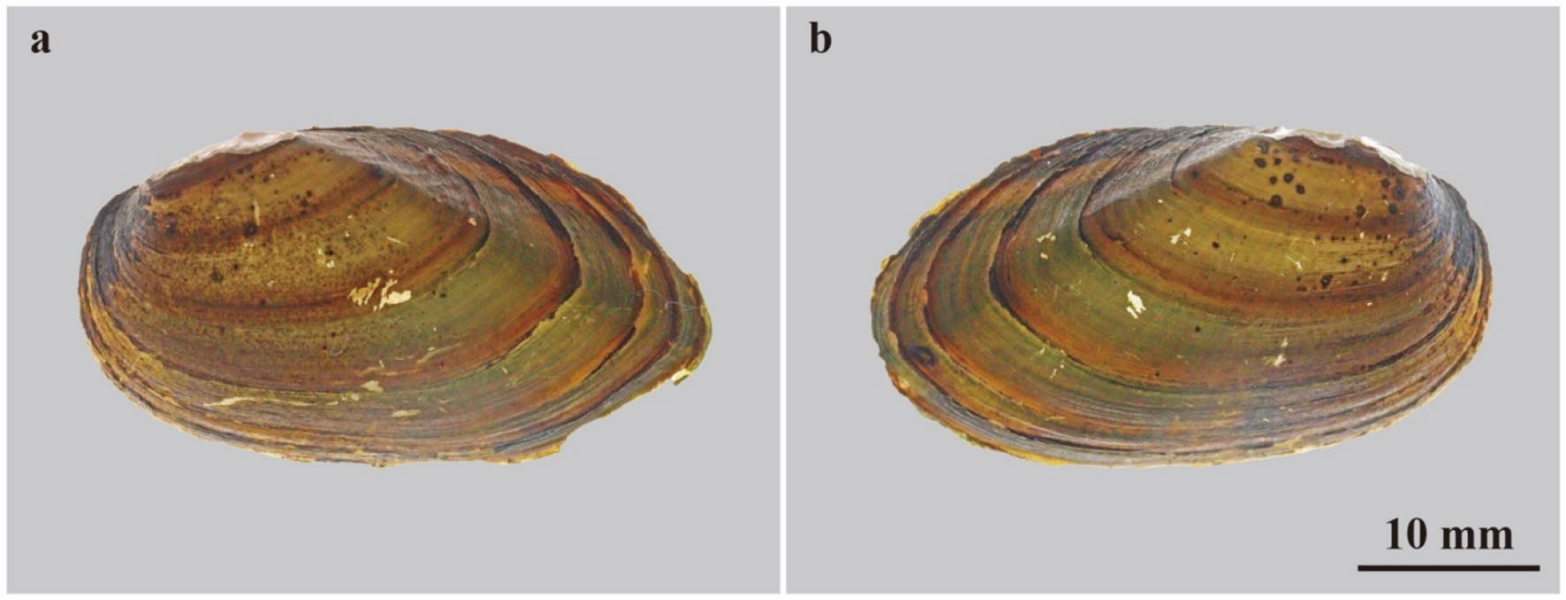
Color patterns of extant freshwater bivalves *Nodularia douglasiae*. (**a**,**b**) FPDM-I-0002915 from Lake Biwa, Shiga, Central Japan. (**a**) Left valve under transmitted light; (**b**) right valve under transmitted light.

**Figure 4:**
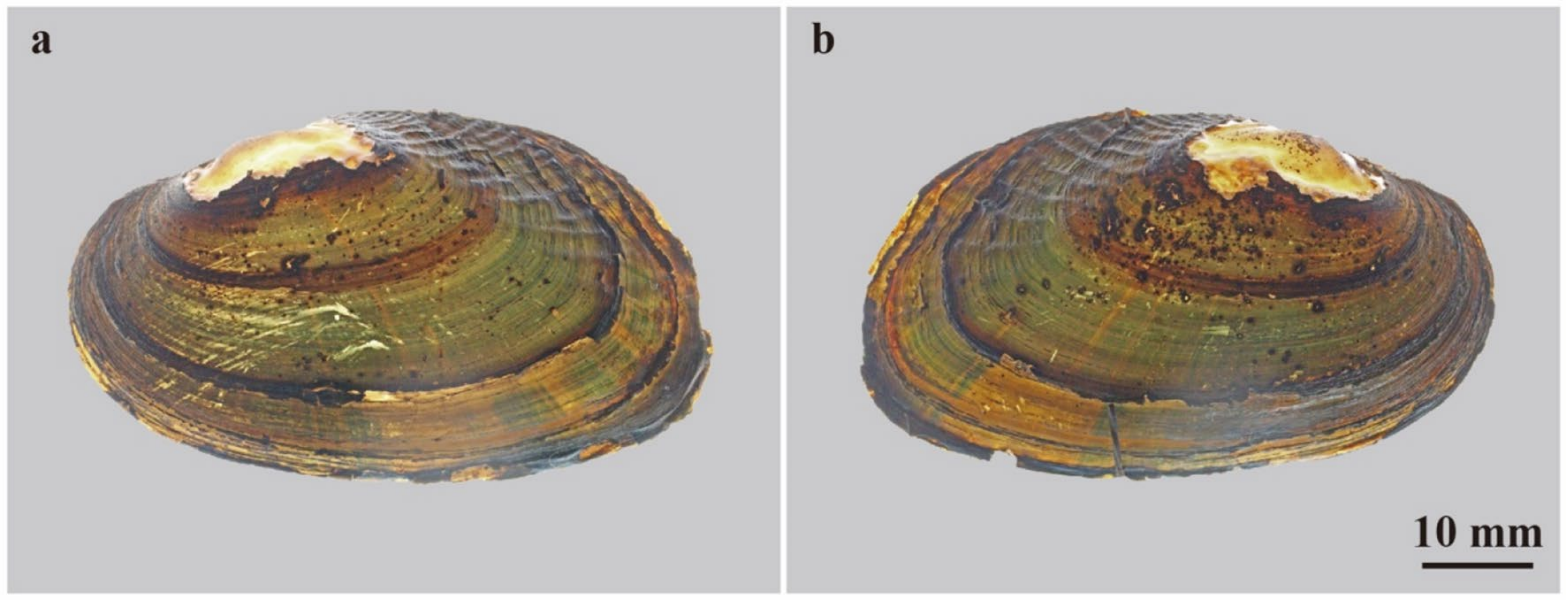
Color patterns of extant freshwater bivalves *Obovalis omiensis*. (**a,b**) FPDM-I-0002921 from Ise, Mie, central Japan. (**a**) Left valve under transmitted light; (**b**) right valve under transmitted light.

**Figure 5:**
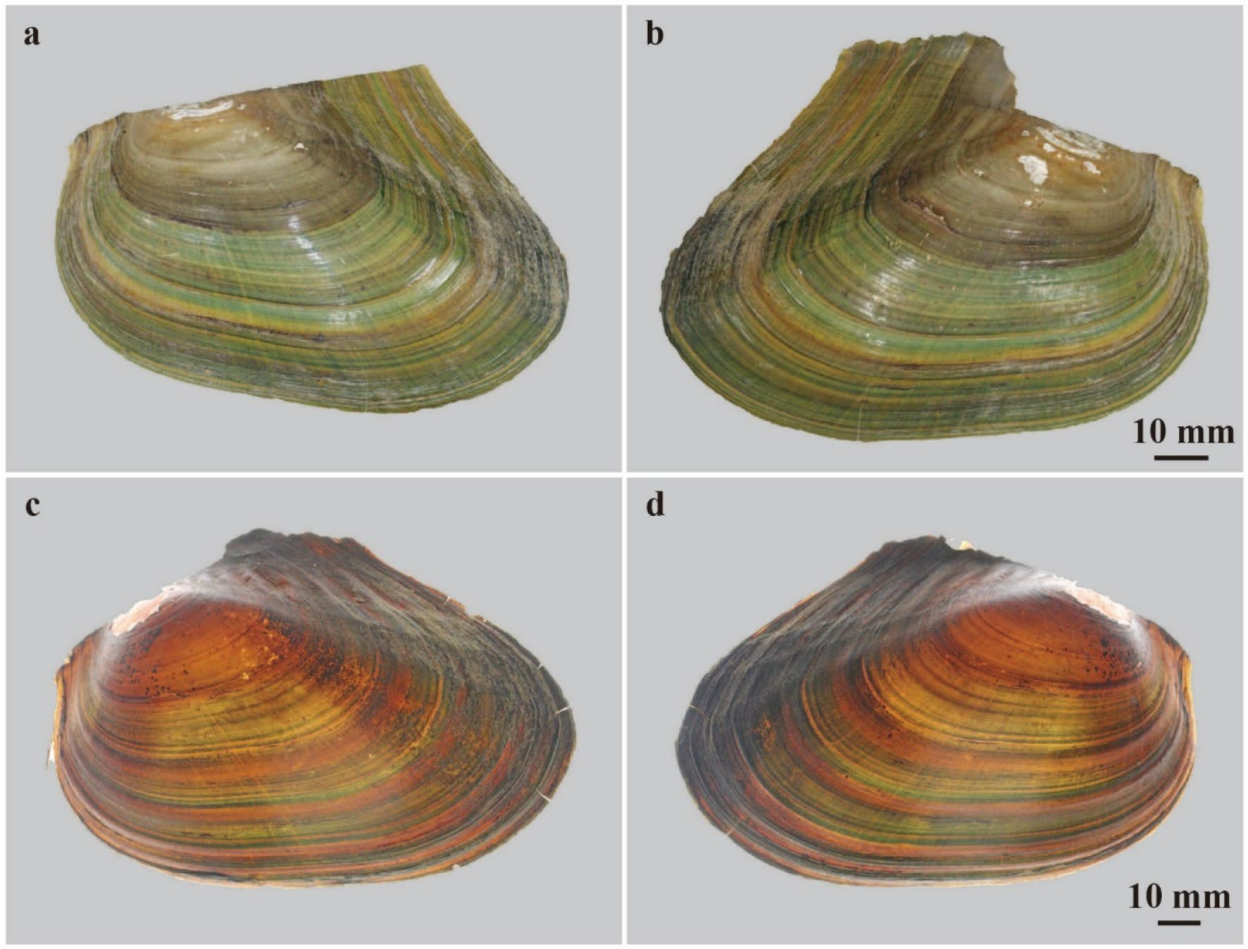
Color patterns of extant freshwater bivalves *Cristaria plicata*. (**a**,**b**) Juvenile individual, FPDM-I-0002911 from Lake Biwa, Shiga, Central Japan. (**a**) Left valve under normal right; (**b**) right valve under normal light. (**c,d**) adult individual, FPDM-I-0002916 from Lake Teganuma, Chiba, eastern Japan. (**c**) Left valve under transmitted light; (**d**) right valve under transmitted light.

Among extant freshwater bivalves, similar color patterns were observed in the Order Unionida (Figs. 2, 3, 4, 5). Patterns can be classified into two types. One type bears four to five, dark green to greenish-brown colored bands that are 2–3 mm wide along the growth lines. The other exhibits bands with various widths and five to twenty dark green to greenish-brown colored rays from the umbo, part of which is bundled to form an approximately 10 mm wide color band. Some unionids are equipped with both types of color bands without any shell ornamentation (*Sinanodonta* and *Cristaria*, Figs. 2, 5, Extended Data Figs. 3 and 4). In contrast, the genera with shell ornamentation, such as plications and wrinkles (*Lanceolaria*, *Nodularia*, and *Obovalis*) lack rays or exhibit them obscurely in the anterior position of the shells (Figs. 3 and 4, Extended Data Figs. 1, 2, 5, 6). In all these taxa, juveniles tended to exhibit brighter and more distinct color patterns than adults.

## Discussion

### Remarks on the residual color patterns in the Kitadani Freshwater Bivalves

Residual color patterns in the form of visible pigmentation on fossil molluscan shells are generally uncommon^2,3^. In the Paleozoic to Mesozoic fossil records, the color patterns were limited to marine species^3^, which are preserved as black to dark-colored bands running on the shell surface as melanin pigments^20,21^. The black to dark-colored stripes on the shells of the Kitadani Freshwater Bivalves resemble the color patterns in some extant freshwater bivalves, suggesting that the dark bands are residual color patterns remaining as melanin pigments. Consequently, the Kitadani Freshwater Bivalves represents the oldest and second fossil record of residual color patterns among fossil freshwater bivalves.

The residual color patterns of the Kitadani Freshwater Bivalves resemble the color patterns of extant freshwater bivalves in terms of width, number, and distribution of the colored bands. Both the Kitadani Freshwater Bivalves and extant freshwater bivalves examined in this study consist of two types of color patterns: stripes along the growth lines and radial rays tapered toward the umbo. Notably, the former pattern is similar among all the species examined, as it forms in the peripheries of prominent growth lines occurring periodically. In the latter pattern, however, the morphology and distribution of the bands are slightly different between the Kitadani Freshwater Bivalves and the extant species. The Kitadani Freshwater Bivalves exhibits relatively distinct and wide radial rays running roughly parallel to the lengths of the sculpture elements (radial plications and/or wrinkles), while the extant species bear obscure and fine radial rays running diagonally to the lengths of the sculpture elements. Nonetheless, the taxa with V-shaped sculpture elements (wrinkles, ribs or arranged nodules) lack or bear ambiguous radial rays, whether extant (e.g., *Triplodon* spp., *Indochinella* spp. and *Tritogonia* spp.)^13,15,22^ or extinct (†*Trigonioides tetoriensis*).

### Hypothesis I: phylogenetic constraints

The resemblance of the color patterns between the Kitadani Freshwater Bivalves and the extant unionids possibly resulted from the phylogenetic constrains. Each of the three species of the Kitadani Freshwater Bivalves belongs to a separate family (†*Trigonioides tetoriensis*: †Trigonioididae, †*Plicatounio naktongensis*: †Plicatounionidae, and †*Matsuomtoina matsumotoi*: †Pseudohyriidae) in the order Trigoniida^19^. Trigoniida in turn, forms the subclass Palaeoheterodonta with Unionida^23^. This raises a possibility that the color patterns observed in the Kitadani Freshwater Bivalves and the extant unionids is inherited from their most recent common ancestor. In other words, these color patterns, stripes along the growth lines and radial rays tapered toward the umbo, may be the apomorphy for Palaeoheterodonta. In fact, some extant trigoniid species belonging to *Neotrigonia* exhibit color pattern similar to those in the Kitadani Freshwater Bivalves and extant unionids in this study (e.g. *Neotrigonia margaritacea*)^1^.

Interestingly, the coloration of color patterns is quite different between unioniids (green to blue colorings) and trigoniids (red to yellow colorings), and the oldest known color patterns of the Palaeoheterodonta (*Myophorella nodulosa*, a marine species of Trigoniida from the Oxfordian of the Early Jurassic) appears different (concentric rows of patches)^10^ from those of the Kitadani Freshwater Bivalves or the extant unioniids. These observations suggest that colorations evolved independently, in contrast to the color patterns, between Trigoniida and Unionida, and that Trigoniida more diverse color patterns than Unionida did in the Palaeoheterodont evolutionary history. Although further examination of the fossil record for the residual colors and color patterns in Palaeoheterodonta is essential, it is plausible that the habitat differences may have caused such discrepancy in the colorations and color patterns between Trigoniida (mainly marine) and Unionida (freshwater) in spite of the phylogenetic constrains.

### Hypothesis II: convergent evolution

The other possible interpretation of the color pattern similarity between the Kitadani Freshwater Bivalves and extant Unionida, is the convergent evolution. One potential factor that may have caused this convergent evolution of the color patterns is an adaptation to their habitats. In general, much of the convergent evolution in animals occurs through the morphological evolution in response to their habitats^24^. Similarly in mollusks, shell colors and their patterns are generally influenced by their habitats^2,6,25^. Considering marine mollusks, the shell colors and their patterns have great diversity due to varying habitat environments, especially in coral reeves that exhibit various colors and complex ecosystem^2,6^. Conversely, in the freshwater ecosystem, the environmental colors are relatively monotonous with rocks, sand, mud, and green algae^8^, and such habitat conditions are likely indifferent between the Mesozoic and Cenozoic. As a result, the freshwater bivalves retained simple and monotonous color patterns for adapting to such environments throughout their evolution.

Another conceivable factor to explain the convergent evolution in the color patterns of the studied freshwater bivalves is the selection pressure by visual predators. In general, the shell colors and their patterns in bivalves act as camouflages against the predators^2,7,8,26–28^. Previous studies have demonstrated that extant freshwater bivalves are preyed upon by crayfish, fish, birds, reptiles, and mammals^29,30^. Because shell colors in freshwater bivalves tend to be greenish, such colors may be an adaptation against visual predators for blending into the freshwater sediments on which abundant greenish phytoplanktons occur^2,8^. Therefore, the evolutionary conservatism in color patterns of freshwater bivalves may result from camouflages into freshwater microenvironments, which has been advantageous against visual predators since the late Early Cretaceous.

The above discussion assumes that the visual predators of freshwater bivalves remained similar for at least 120 million years. Which animals could have been potential threads to the Kitadani Freshwater Bivalves, and, in turn, the Early Cretaceous freshwater bivalves? Among the extant visual predators of the freshwater bivalves, those whose lineages were present in the Early Cretaceous include crustaceans (especially brachyuran decapoda^31^), fish, lizards, turtles, crocodiles, birds, and mammals. Among them, the fossil record of durophagous lizards and mammals can be traced back only to the Late Cretaceous^32,33^. Conversely, lines of fossil evidence suggest that some fish^34,35^, turtles^36^, and crocodiles^35^ fed on molluscan invertebrates during the Early Cretaceous, and the Kitadani Freshwater Bivalves indeed occurs with abundant lepisosteiform scales, testudinate shells and crocodile teeth. Additionally, at least one Early Cretaceous avian species with crustacean gut contents can be attributed to the durophagous diet^37^, and the Kitadani Formation has yielded avialan skeletal remains^38^, and footprints^39,40^. Therefore, fish, turtles, crocodiles, and birds are likely candidates for visual predators of the Early Cretaceous freshwater bivalves, and have remained so until present. Additionally, while crustaceans have not been identified in the Kitadani Formation, they flourished in the Early Cretaceous and their remains occur with the fossil freshwater bivalves of the time elsewhere^31^. Thus, crustaceans may have also played a role as visual predators of the freshwater bivalves since the Early Cretaceous.

In addition to the crustaceans, fishes, turtles, crocodiles and birds, the visual predators of the Early Cretaceous freshwater bivalves likely include extinct lineages. For example, some pliosauroid plesiosaurs are suggested as being durophagous^34^, although the freshwater members of the group are considered endemic^41^ and less likely to be a major thread to the Early Cretaceous freshwater bivalves. Another extinct candidate is non-avian dinosaurs. Ornithischians are suggested to have possessed a dietary flexibility including the durophagy. For instance, well-preserved hadrosaurid coprolites from the Late Cretaceous of Montana, U.S.A. include sizeable crustaceans and mollusks, possibly suggesting that the Cretaceous freshwater mollusks were consumed by these herbivorous dinosaurs^42^. In addition, some basal ceratopsian psittacosaurids are hypothesized for the durophagy based on the predicted large bite force in the caudal portion of the toothrow^43^. Among saurischians, some oviraptorosaurian theropods are indicated to consume mollusks with hard shells based on their mandibular features^44^. While hadrosaurids, psittacosaurids, and oviraptorosaurians have not been identified in the Kitadani Formation, psittacosaurids, and oviraptorosaurians are common elsewhere in the Early Cretaceous of East Asia^45,46^, and hadrosauroid *Koshisaurus* is present in the formation^47^. Because dinosaurs occupied a niche of large terrestrial predators throughout the Mesozoic, they may have acted as one of major mollusk consumers in absence of large lizards and mammals in the Early Cretaceous ecosystem. Thus, the predation pressure by visual predators to the freshwater bivalves in the Early Cretaceous is likely similar to that in the present. Consequently, one of evolutionary adaptations of the freshwater bivalves against such pressure has remained to camouflage in the phytoplankton-rich sediments, leading to the long-term evolutionary conservatism of their color patterns.

## Conclusions

Our study provides evidence for potential phylogenetic constraints in the shell color patterns in the freshwater bivalves, namely Trigoniida and Unionida. Alternatively, our study exemplifies possible convergent evolution that occurred at least 120 million years apart in the evolutionary history of these taxa. The convergence may be promoted by monotonous habitats typical of freshwater ecosystems. Another possible explanation to this convergent evolution is the predation pressure by visual predators like crustaceans, fishes, turtles, crocodiles and dinosaurs (replaced by birds and mammals today), and the evolutionary adaptation against such pressure to camouflage in the freshwater sediments. To further test our hypotheses about the evolution of the color patterns in the freshwater bivalves, it is mandate to accurately evaluate the selective pressures that cause the adaptation of the color patterns in modern taxa. Nonetheless, our study provides an opportunity to explore the mechanisms that determine color patterns of freshwater mollusks and represents a milestone to resolve their adaptive evolution in the color patterns.

## Methods

The studied specimens for extant freshwater bivalves are deposited at the Fukui Prefectural Dinosaur Museum (FPDM) (Extended Data Table 1). For thin-shelled species (e.g., *Sinanodonta* spp.), normal light was transmitted under the shells for identifying color patterns. Photographs, except for transmitted pictures, were taken with lighting from the northwest, as shown in Figs. 2, 3, 4, 5 and Extended Data Figs. 1–6, using a Canon Eos Kiss X10 with SP AF60mm F/2 Di II LD [IF] MACRO 1:1 and Canon EF-S18-55 mm F3.5–5.6 IS II stopped down to f/13.

Fossil freshwater bivalves were collected from the Kitadani Dinosaur Quarry, Katsuyama, Fukui, central Japan, where the Lower Cretaceous Kitadani Formation (Aptian) of the Tetori Group crops out. Among approximately 6000 bivalve individuals collected from the quarry, we selected the best preserved individuals for analyzing color patterns, resulting in 17 specimens (Extended Data Table 2). The specimens were mechanically prepared using powerful flying pneumatic scribes including HW-65 with a pointed 3 mm tips and HW-322 with a 1.3 mm needle (German Engineered Precision Tools, Tethys, 1-73-5 Beppu, Mizuho, Gifu, Japan). Thin sediments, and diagenetic minerals on the shell surface were removed using a sand blasting tool KRANTZ sandblaster 70-250 µm W1625 with reduced iron powder #150 (75–150 µm in diameter with a new Mohs hardness of 4.5; Fuji Manufacturing) adjusted to 0.7–0.8 MPa. After blasting, apricot powders #150 (75–150 µm in diameter with a new Mohs hardness of 3.5; Fuji Manufacturing) adjusted to 0.7–0.8 MPa was used to remove fine sediments and minerals without damaging the shell. After preparation, the fossil specimens were photographed using a Canon Eos Kiss X10 with a SP AF60mm F/2 Di II LD [IF] MACRO 1:1 lens using two methods: whitening for shell ornamentation and water-immersion for residual color patterns. Whitening photography was conducted for Extended Data Figs. 7, 8b,d, 9b,d, 10b,d, 11, 12b,d, 13b,d, 14b,d, 15b, 16, 17b,d, 18b,d, 19b,d by letting the shell surface coated with ammonium chloride, and lightning from the northwest to enhance contrast. Residual color patterns of the fossil specimens were imaged by immersing them in water and photographed with lightning sourced from the northwest, and adjusted so that the photographs of the light directions were identical between the water-immersion and whitening photography.

Transmitted, whitening, and water-immersed images were post-processed using Adobe Photoshop 2020, first applying the ‘sharpen more’ and ‘sharpen’ functions, followed by background removal. Minor adjustments were occasionally made to the exposure. The high-resolution images were down-sampled using Adobe illustrator 2021 to lower-resolution Tiff files for use in the plates.

Reconstruction drawings of residual color patterns in fossil freshwater bivalves (Fig. 1d–f) were prepared using Adobe Illustrator 2021 based on high-resolution images. The drawings were applied to the CMYK color model.

## Supplementary Information

Supplementary Information.

## Data Availability

All data generated or analyzed during this study are included in this published article [and its supplementary information files S1].
