# Supplementary material for: Case study of the convergent evolution in the color patterns in the freshwater bivalves
Source: Sci Rep. 2022 Jul 13;12:10885. doi: 10.1038/s41598-022-14469-3 (PMC9279500; doi:10.1038/s41598-022-14469-3)
Supplement: Supplementary file 1 — Supplementary Information. [file 41598_2022_14469_MOESM1_ESM.docx]

**SUPPLEMENTARY INFORMATION**

**Case study of the convergent evolution in the color pattern in the freshwater bivalves**

Kaito Asato^1*^, Kentaro Nakayama^1^ and Takuya Imai^2^

*^1^Fukui Prefectural Dinosaur Museum, 51-11 Terao, Muroko, Katsuyama, Fukui 911-8601, Japan*

*^2^Institute of Dinosaur Research, Fukui Prefectural University, Japan*

Correspondence and requests for materials should be addressed to K.A.

^*^email: [k-asato@dinosaur.pref.fukui.jp](mailto:k-asato@dinosaur.pref.fukui.jp)

**
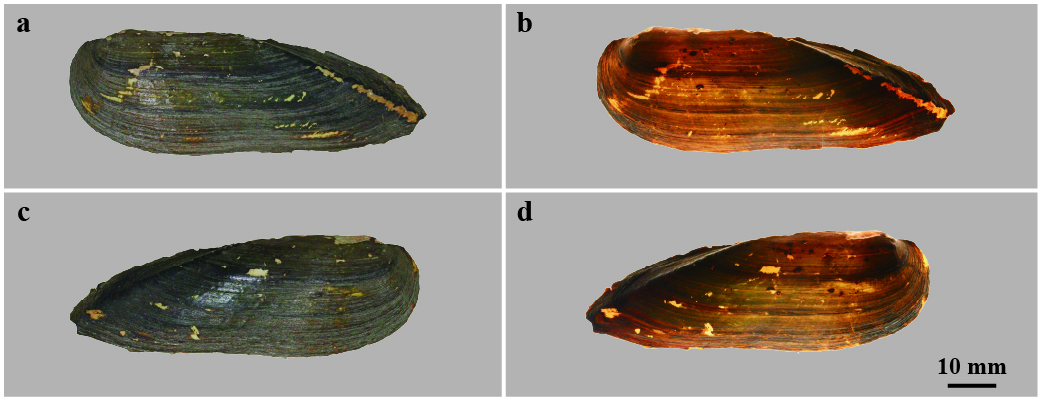
Extended Data Figure 1. Color patterns of extant freshwater bivalves. a–d**, *Lanceolaria oxyrhyncha*, FPDM-I-0002920 from Lake Biwa, Shiga, Central Japan. **a**, left valve under normal light; **b**, left valve under transmitted light; **c**, right valve under normal light; **d**, right valve under transmitted light.


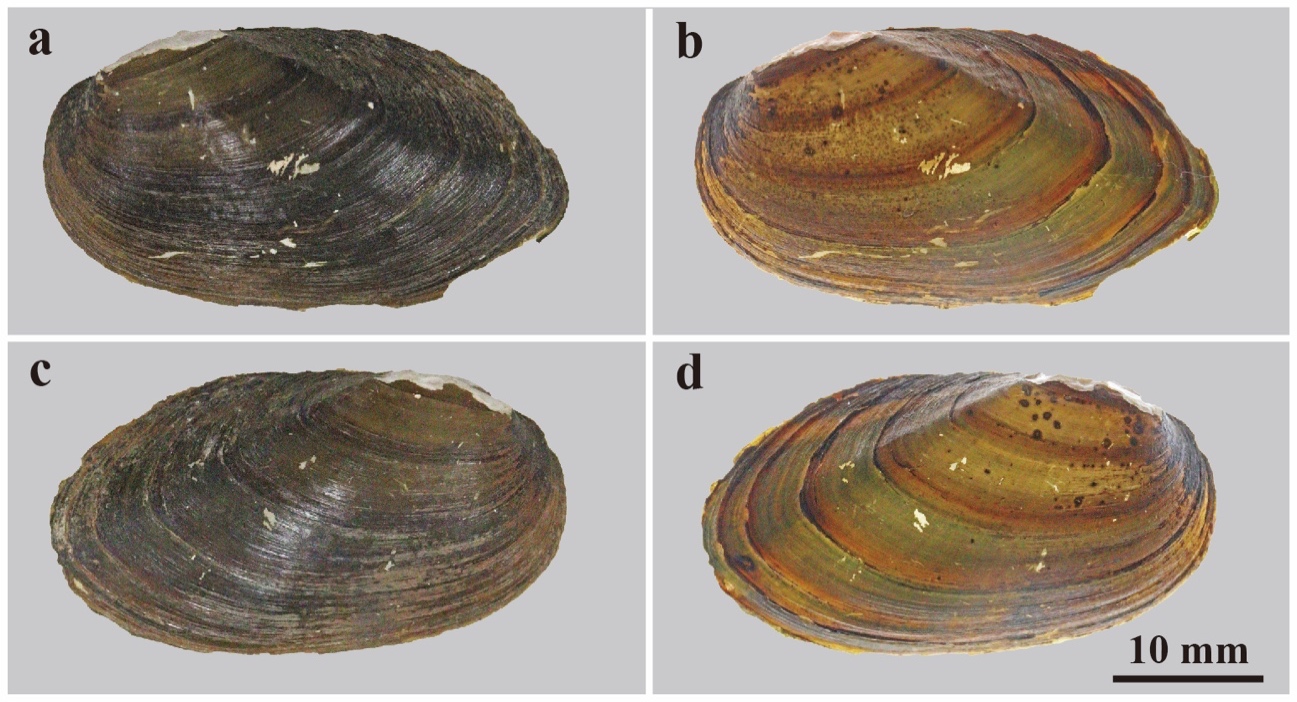
**Extended Data Figure 2. Color patterns of extant freshwater bivalves. a–d**, *Nodularia* *douglasiae*, FPDM-I-0002915 from Lake Biwa, Shiga, Central Japan. **a**, left valve under normal light; **b**, left valve under transmitted light; **c**, right valve under normal light; **d**, right valve under transmitted light.

**
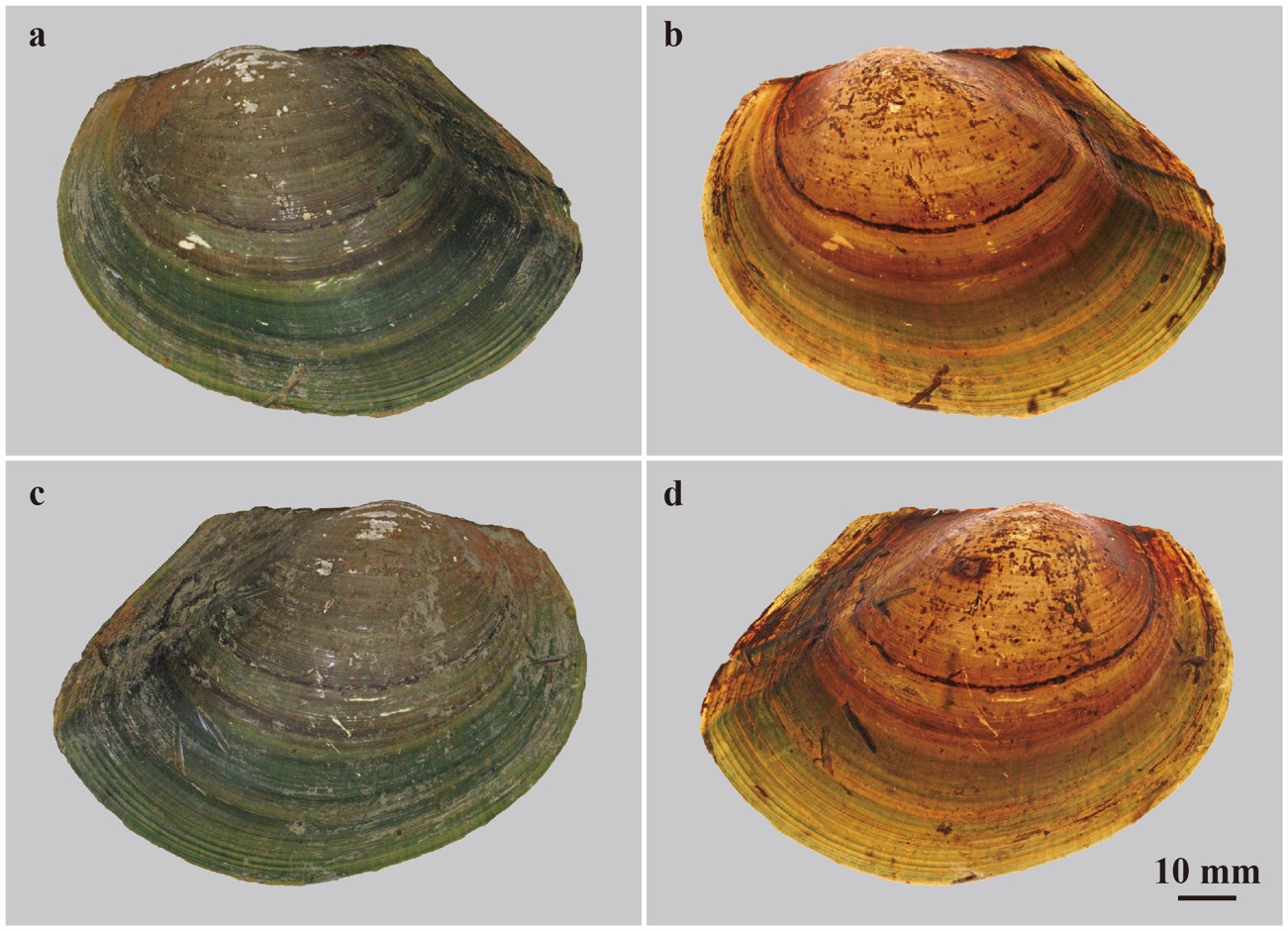
 Extended Data Figure 3. Color patterns of extant freshwater bivalve *Sinanodonta* *calipygos***. **a–d**, FPDM-I-0002924 from Lake Biwa, Shiga, Central Japan. **a**, left valve under normal light; **b**, left valve under transmitted light; **c**, right valve under normal light; **d**, right valve under transmitted light.


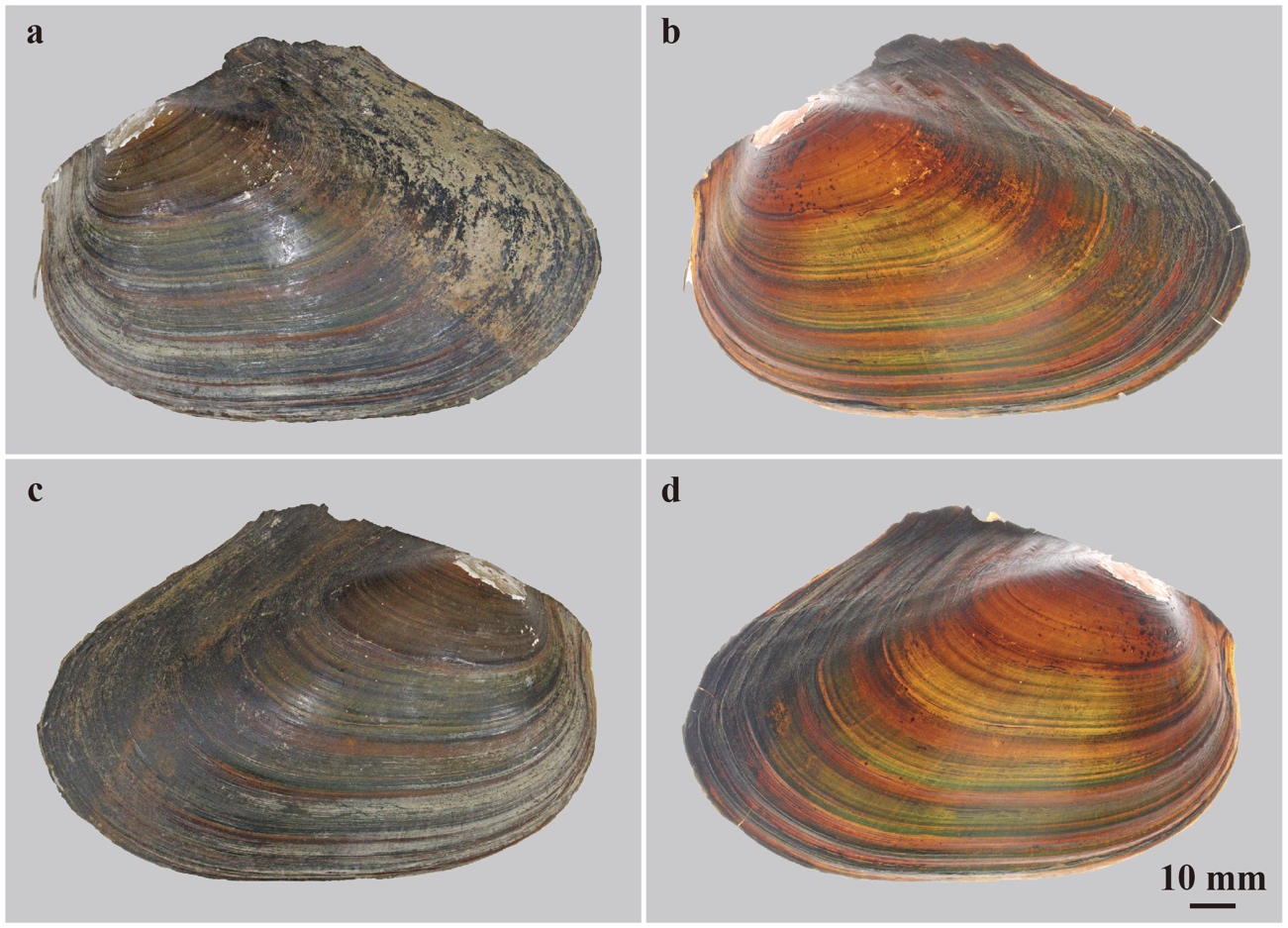
**Extended Data Figure 4. Color patterns of extant freshwater bivalve *Cristaria* *plicata*. a**–**d**, FPDM-I-0002916 from Lake Biwa, Shiga, Central Japan; **c**, left valve under normal light; **d**, left valve under transmitted light; **e**, right valve under normal light; **f**, right valve under transmitted light.


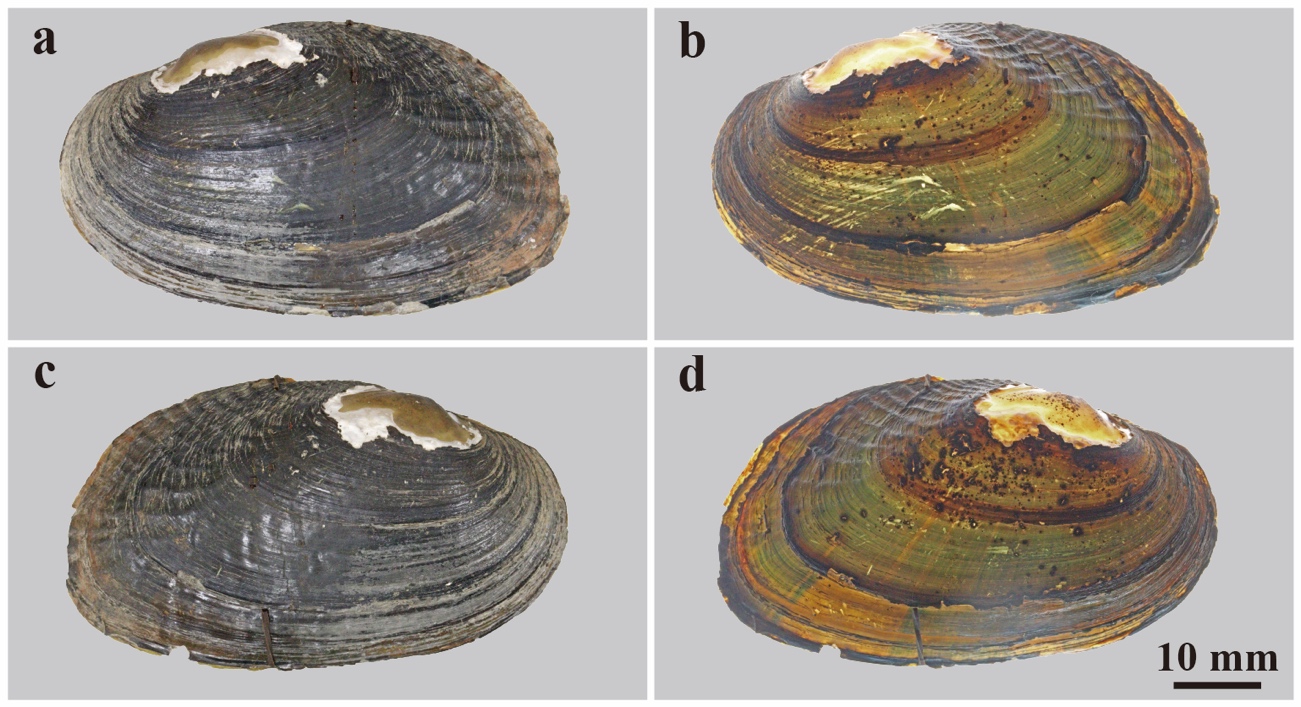
**Extended Data Figure 5. Color patterns of extant freshwater bivalves. a–d**, *Obovalis* *omiensis*, FPDM-I-0002921 from Ise, Mie, Central Japan. **a**, left valve under normal light; **b**, left valve under transmitted light; **c**, right valve under normal light; **d**, right valve under transmitted light.


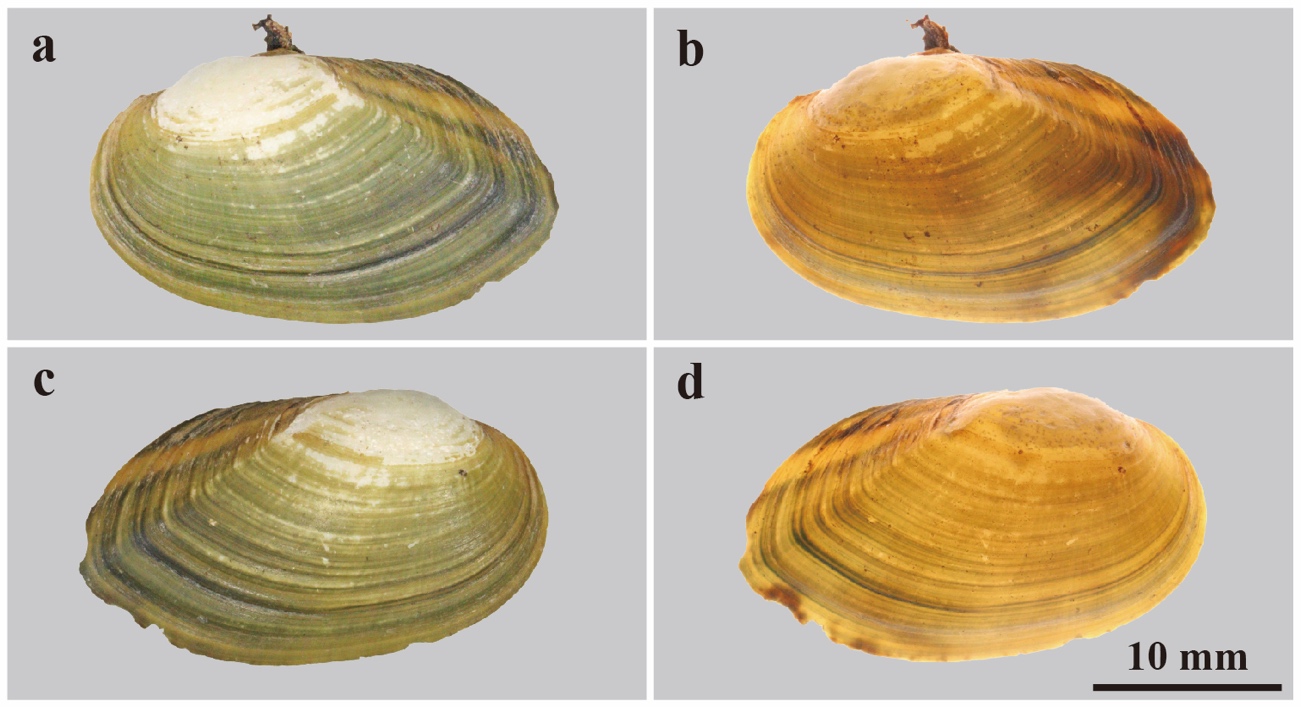
**Extended Data Figure 6. Color patterns of extant freshwater bivalves. a–d**, a juvenile individual of *Nodularia* *douglasiae*, FPDM-I-0002917 from Lake Biwa, Shiga, Central Japan. **a**, left valve under normal light; **b**, left valve under transmitted light; **c**, right valve under normal light; **d**, right valve under transmitted light.


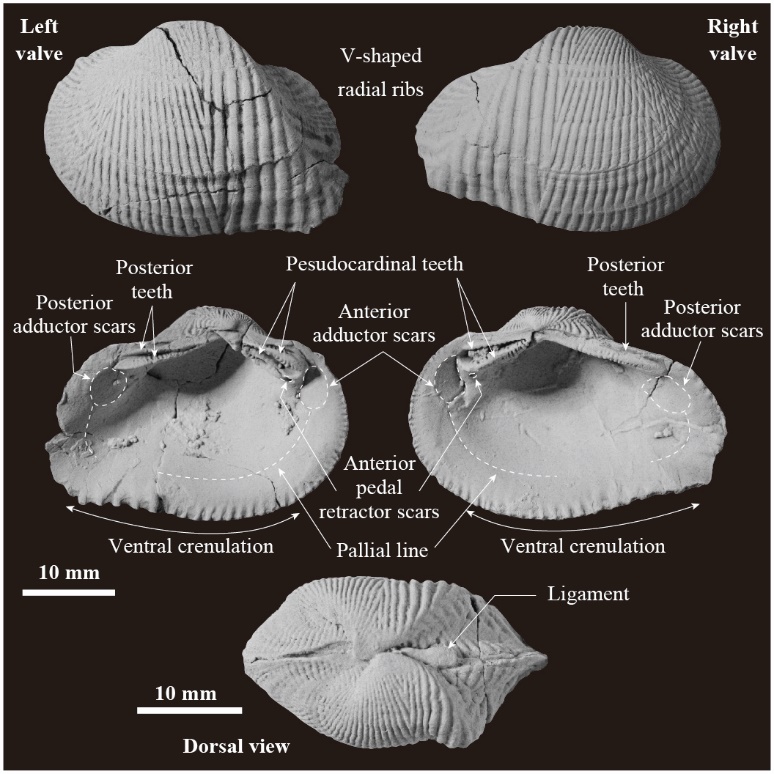


**Extended Data Figure 7. Morphological features of *Trigonioides* (*Wakinoa*) *tetoriensis* from the Kitadani Formation.** External (FPDM-I-3447), internal (FPDM-I-3447), and dorsal (FPDM-I-3443) views were coated with ammonium chloride.

**
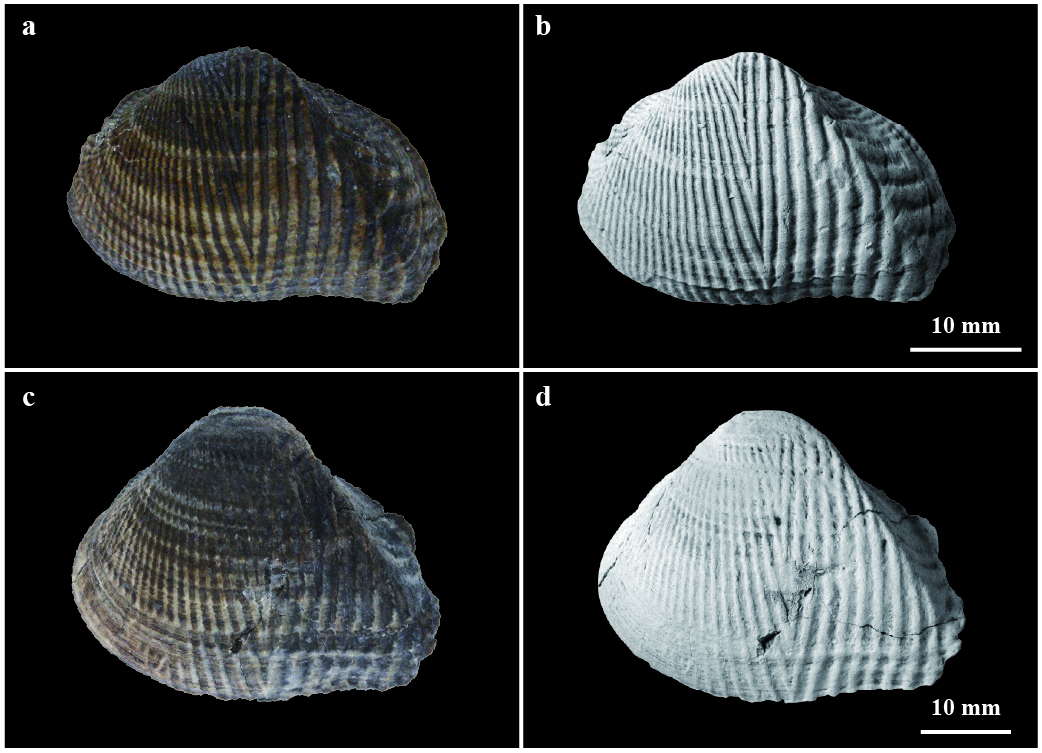
Extended Data Figure 8. Color pattern and shell ornamentation of *Trigonioides* (*Wakinoa*) *tetoriensis*, from the Kitadani Formation. a–b**, FPDM-I-3445, left valve. **a**, water-immersed for color pattern; **b**, whitened for shell ornamentation with ammonium chloride; **c–d**, FPDM-I-3442, left valve; **c**, water-immersed for color pattern; **d**, whitened for shell ornamentation with ammonium chloride.


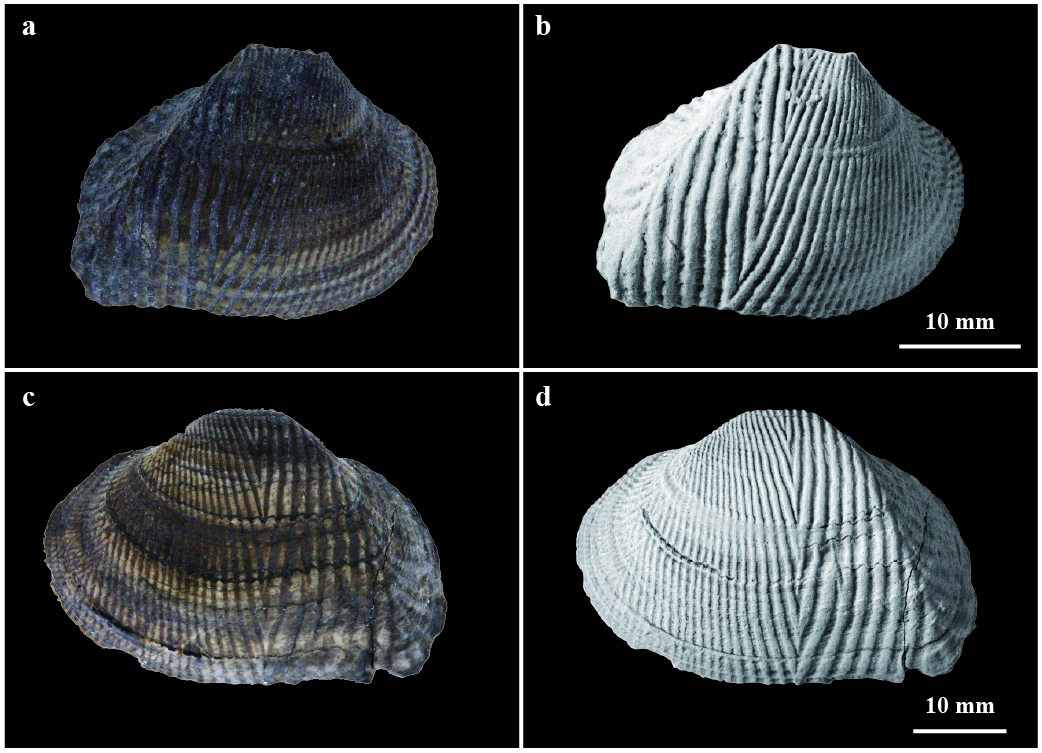
**Extended Data Figure 9. Color pattern and shell ornamentation of *Trigonioides* (*Wakinoa*) *tetoriensis*, from the Kitadani Formation. a–b**, FPDM-I-3446, right valve. **a**, water-immersed for color pattern; **b**, whitened for shell ornamentation with ammonium chloride; **c–d**, FPDM-I-3444, left valve. **c**, water-immersed for color pattern; **d**, whitened for shell ornamentation with ammonium chloride.

**
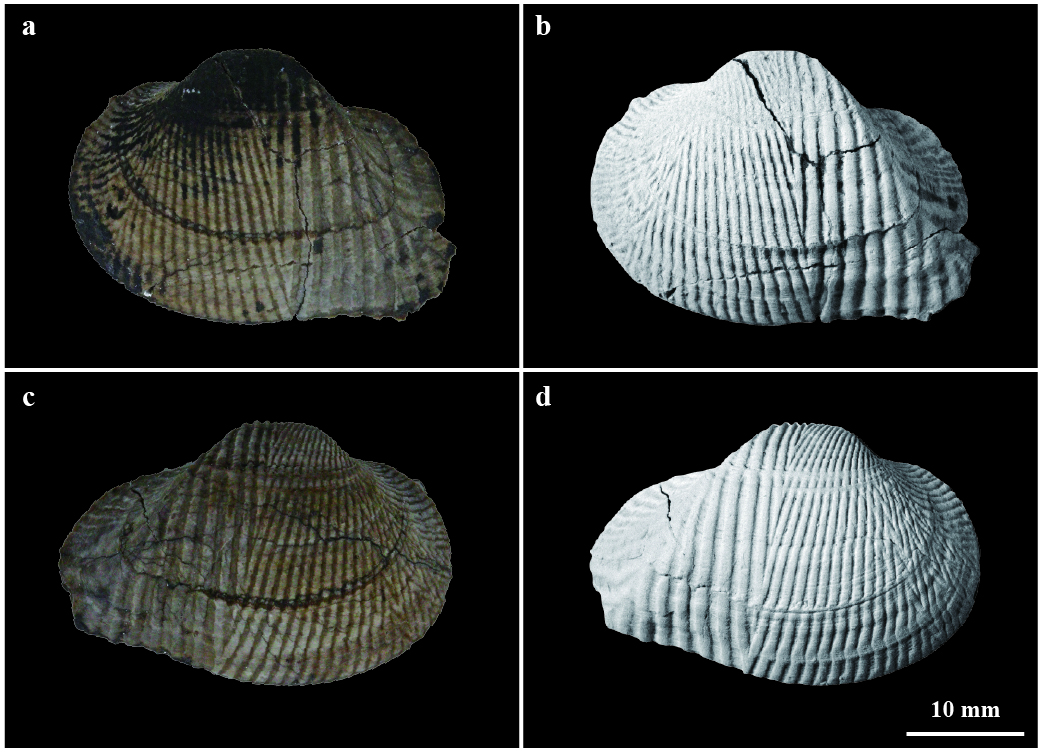
Extended Data Figure 10. Color pattern and shell ornamentation of *Trigonioides* (*Wakinoa*) *tetoriensis*, from the Kitadani Formation.** **a–d**, FPDM-I-3447, articulated valves; **a**, left valve water-immersed for color pattern; **b**, left valve whitened for shell ornamentation with ammonium chloride; **c**, right valve water-immersed for color pattern; **d**, right valve whitened for shell ornamentation with ammonium chloride.

**
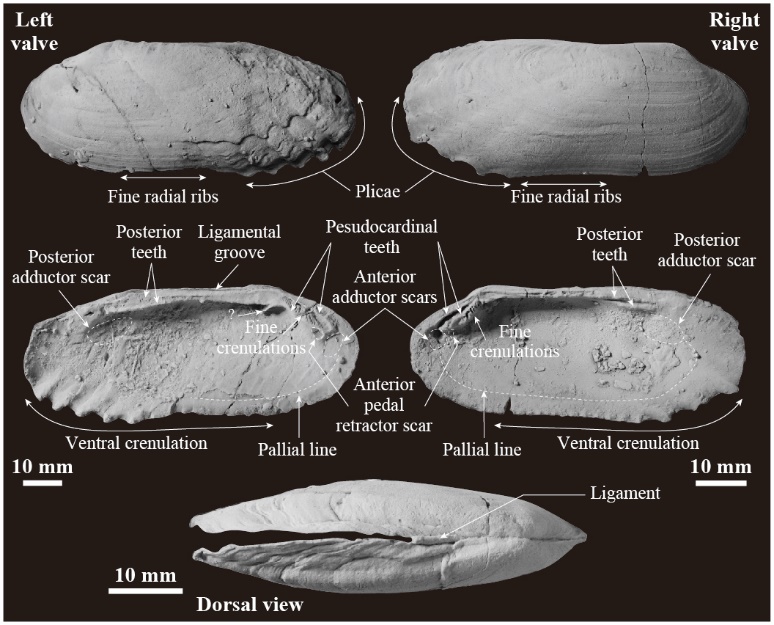
**

**Extended Data Figure 11. Morphological features of *Plicatounio* (*Plicatounio*) *naktongensis* from the Kitadani Formation.** External (FPDM-I-3449 for left valve, and FPDM-I-3452 for right valve), internal (FPDM-I-3449 for left valve, and FPDM-I-3452 for right valve), and dorsal (FPDM-I-3451) views, coated with ammonium chloride.

**
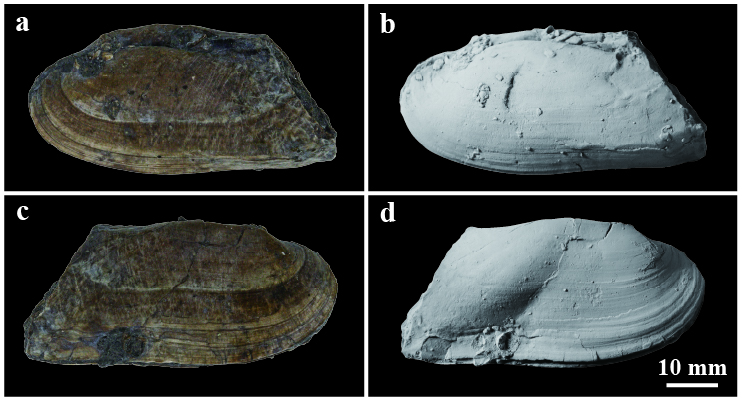
**

**Extended Data Figure 12. Color pattern and shell ornamentation of *Plicatounio* (*Plicatounio*) *naktongensis* from the Kitadani Formation. a–d**, FPDM-I-3450, articulated valves. **a**, left valve water-immersed for color pattern; **b**, left valve whitened with ammonium chloride for shell ornamentation; **c**, right valve water-immersed for color pattern; **d**, right valve whitened with ammonium chloride for shell ornamentation.


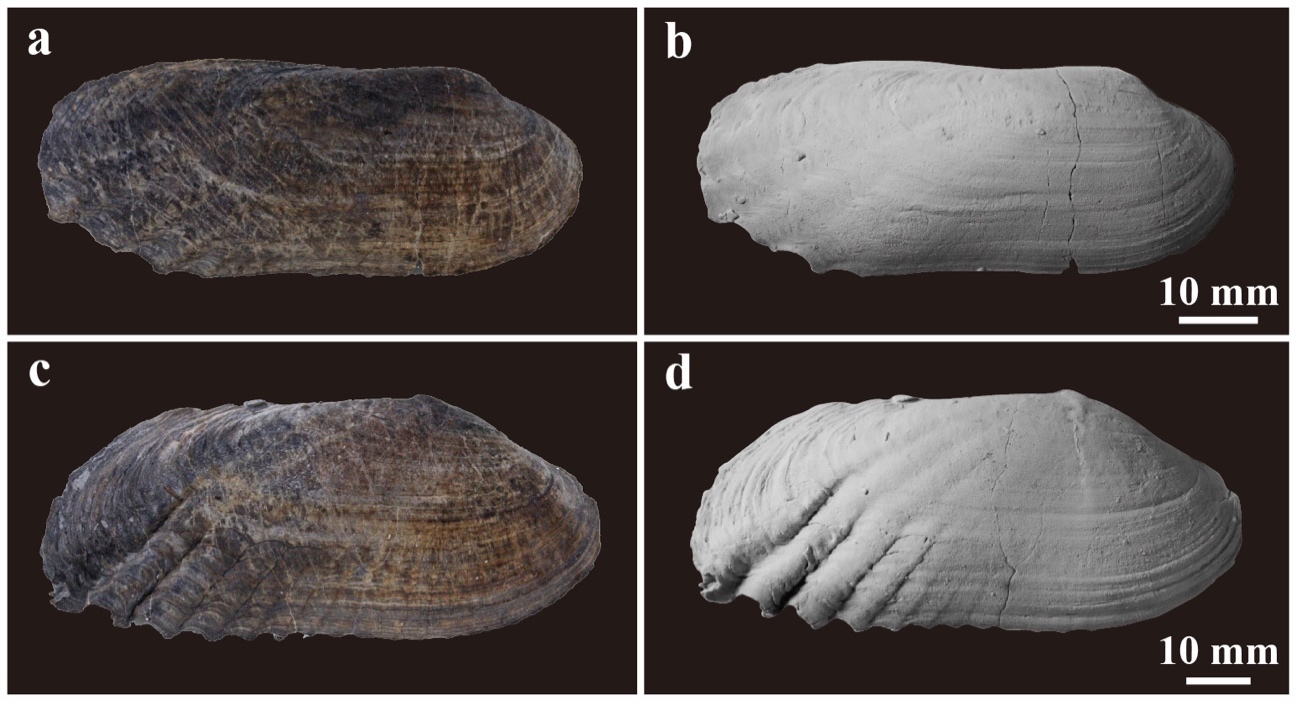
**Extended Data Figure 13. Color pattern and shell ornamentation of *Plicatounio* (*Plicatounio*) *naktongensis* from the Kitadani Formation. a–b**, FPDM-I-3452, right valve; **a**, water-immersed for color pattern; **b**, whitened with ammonium chloride for shell ornamentation; **c–d**, FPDM-I-3453, right valve; **c**, water-immersed for color pattern; **d**, whitened with ammonium chloride for shell ornamentation.

**
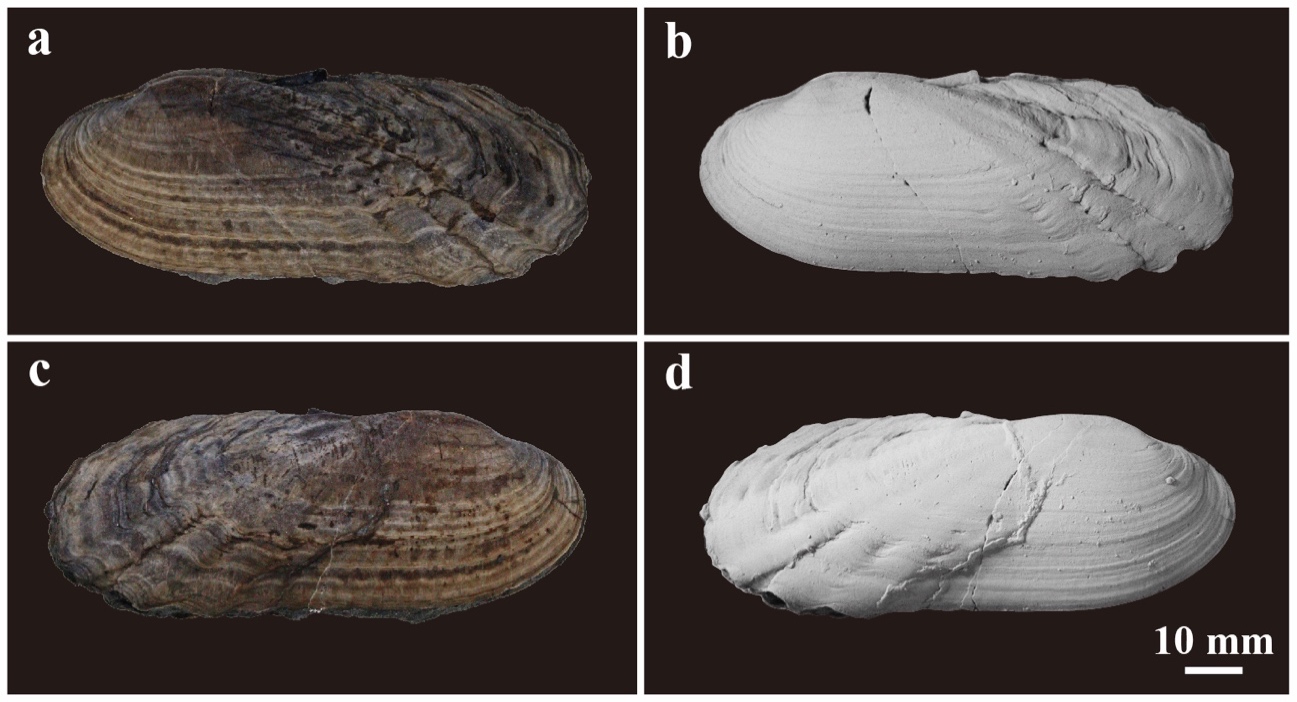
Extended Data Figure 14. Color pattern and shell ornamentation of *Plicatounio* (*Plicatounio*) *naktongensis* from the Kitadani Formation. a–d**, FPDM-I-3451, articulated valves. **a**, left valve water-immersed for color pattern; **b**, left valve whitened with ammonium chloride for shell ornamentation; **c**, right valve water-immersed for color pattern; **d**, right valve whitened with ammonium chloride for shell ornamentation.


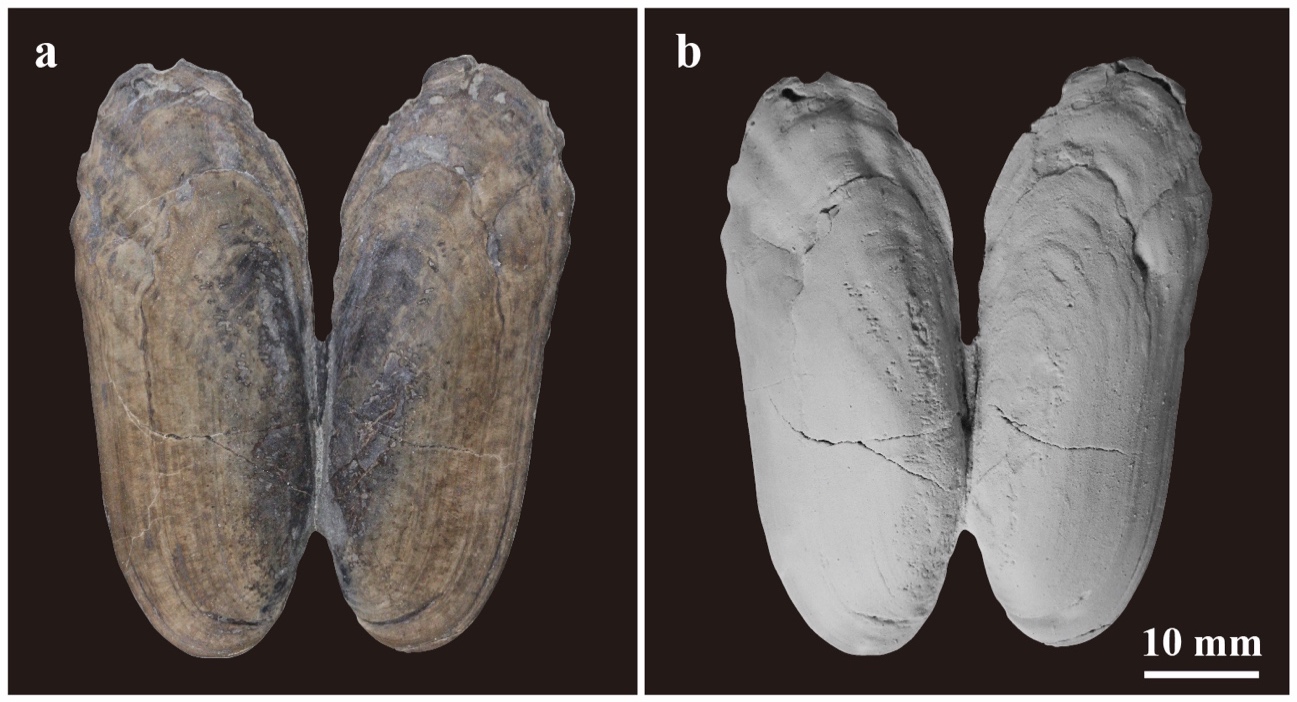
**Extended Data Figure 15. Color pattern and shell ornamentation of *Plicatounio* (*Plicatounio*) *naktongensis* from the Kitadani Formation. a–b**, FPDM-I-3448, butterfly position; **a**, water-immersed image for color pattern; **b**, whitened with ammonium chloride for shell ornamentation.

**
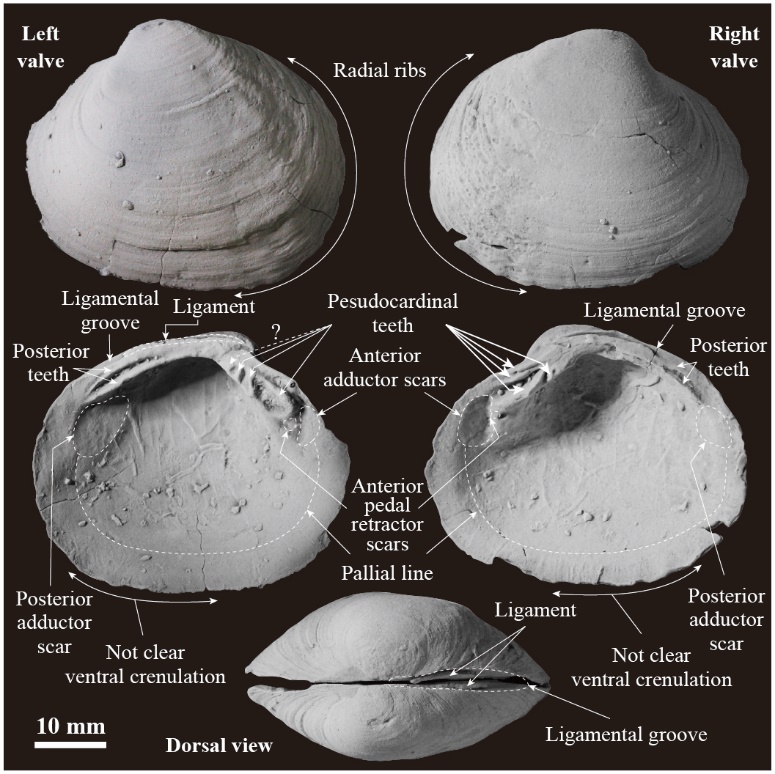
**

**Extended Data Figure 16. Morphological features of *Matsumotoina matsumotoi* from the Kitadani Formation of Tetori Group, Japan.** FPDM-I-3460 was coated with ammonium chloride.

**
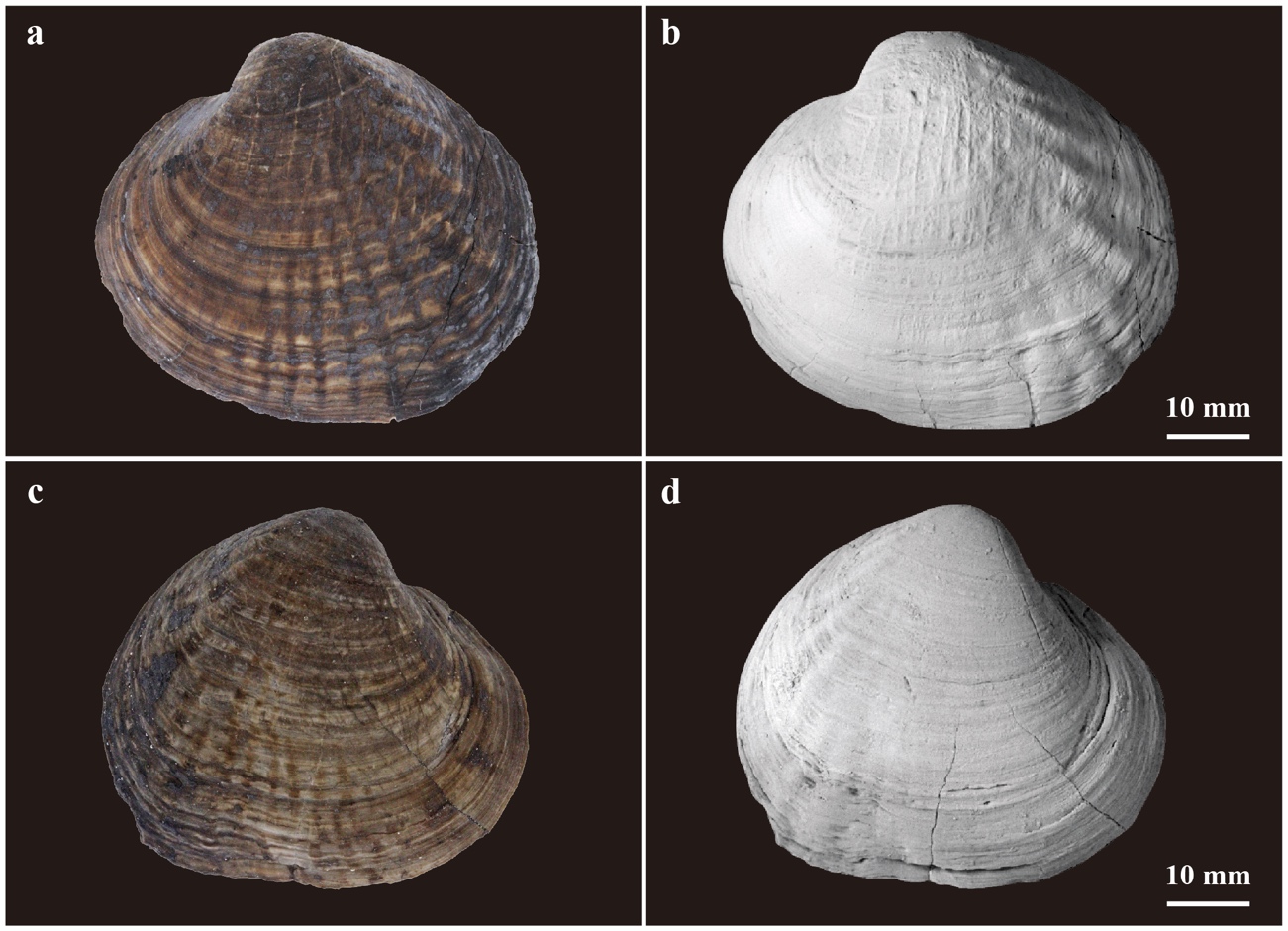
Extended Data Figure 17. Color pattern and shell ornamentation of *Matsumotoina matsumotoi* from the Kitadani Formation.** **a–b**, FPDM-I-3456, left valve. **a**, water-immersed for color pattern; **b**, whitened with ammonium chloride for shell ornamentation; **c–d**, FPDM-I-3457, right valve; **c**, water-immersed for color pattern; **d**, whitened with ammonium chloride for shell ornamentation.


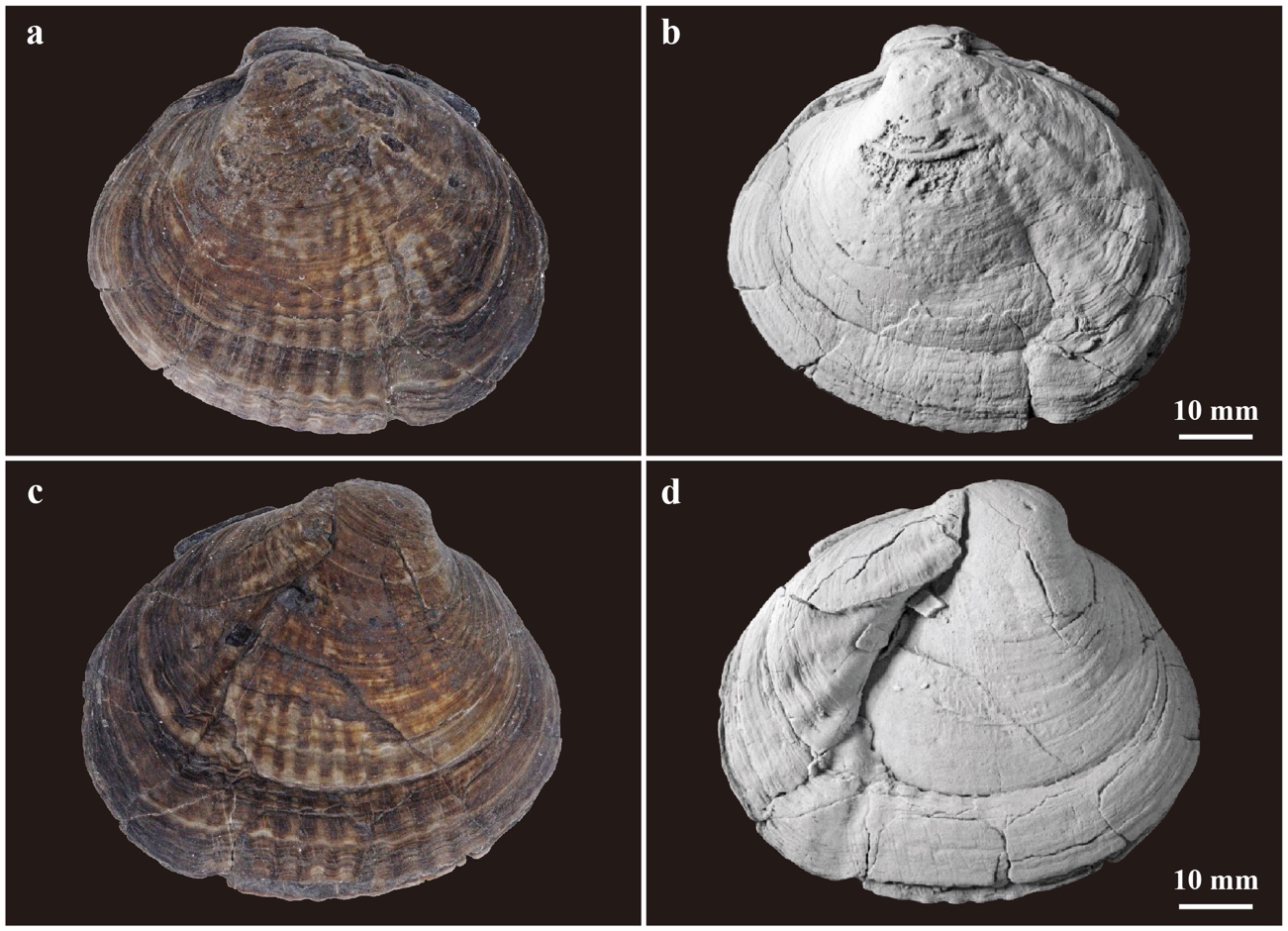
**Extended Data Figure 18. Color pattern and shell ornamentation of *Matsumotoina matsumotoi* from the Kitadani Formation. a–d**, FPDM-3458, articulated valves. **a**, left valve water-immersed for color pattern; **b**, whitened with ammonium chloride for shell ornamentation; **c**, right valve water-immersed for color pattern; **d**, right valve whitened with ammonium chloride for shell ornamentation.

**
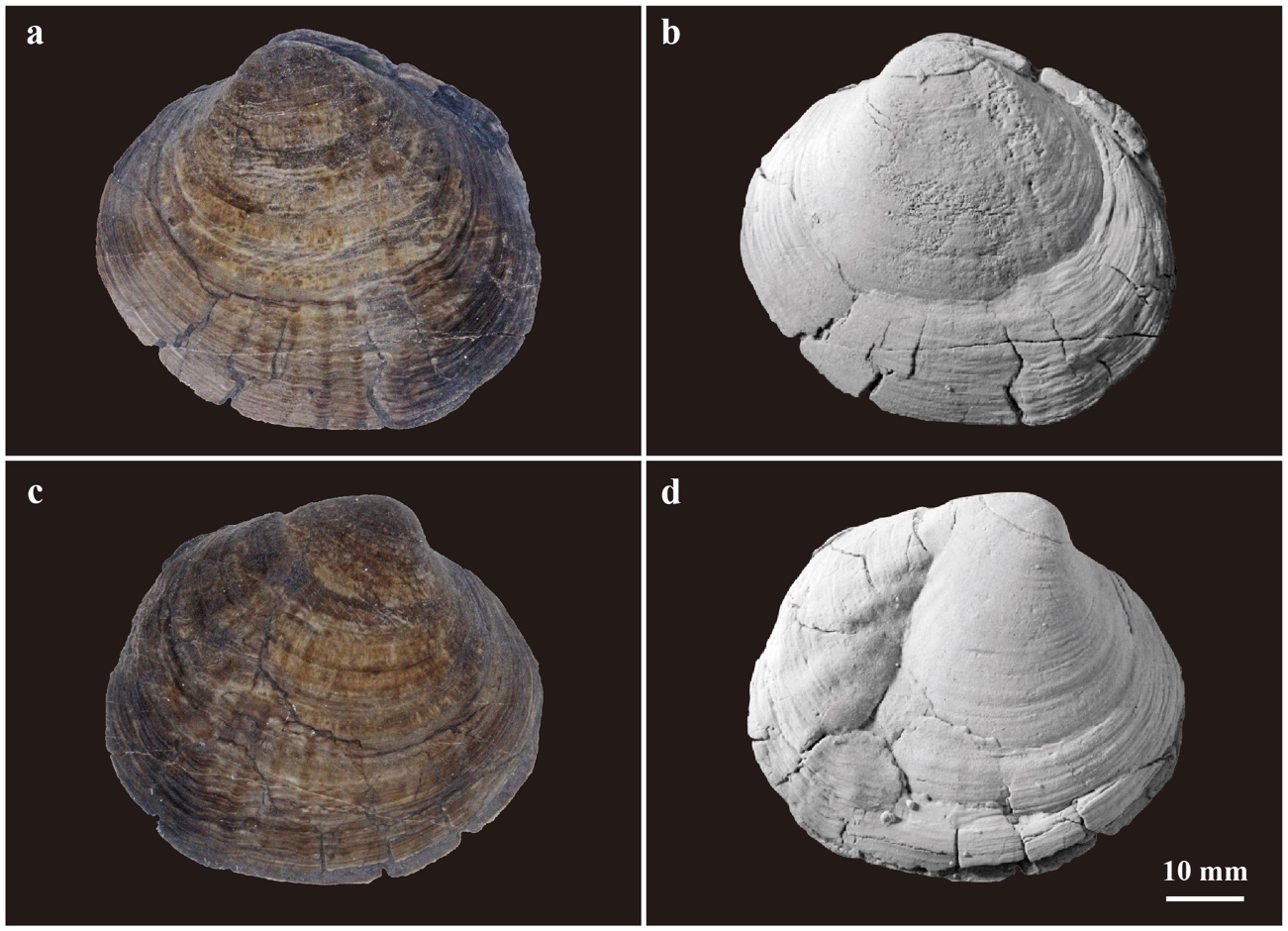
Extended Data Figure 19. Color pattern and shell ornamentation of *Matsumotoina matsumotoi* from the Kitadani Formation of Tetori Group, Japan.** **a–d**, FPDM-I-3459, articulated valves; **a**, left valve water-immersed for color pattern; **b**, left valve whitened with ammonium chloride for shell ornamentation; **c**, right valve water-immersed for color pattern; **d**, right valve whitened with ammonium chloride for shell ornamentation.

**Extended Data Table 1. Extant freshwater bivalves described in this study.**

| Taxon | | Specimen | Locality | Number of Individuals |
| --- | --- | --- | --- | --- |
| Unionidae | *Lanceolaria* *oxyrhyncha* | FPDM-I-0002920 | Lake Biwa, Shiga, Japan | 2 |
|  |  | FPDM-I-0002913 |  | 5 |
|  | *Nodularia* *douglasiae* | FPDM-I-0002915 |  | 7 |
|  |  | FPDM-I-0002917 |  | 2 |
|  | *Sinanodonta* *calipygos* | FPDM-I-0002924 |  | 3 |
|  | *Cristaria* *plicata* | FPDM-I-0002911 |  | 2 |
|  |  | FPDM-I-0002916 |  | 3 |
|  | *Obovalis* *omiensis* | FPDM-I-0002921 | Ise, Mie, Japan | 2 |

**Extended Data Table 2. Fossil freshwater bivalves described in this study from the Kitadani Formation cropping out in the Kitadani Dinosaur Quarry. Each specimen contains a single sample.**

| Taxon | | Specimen | Preservation |
| --- | --- | --- | --- |
| Trigonioididae | *Trigonioides* (*Wakinoa*) *tetoriensis* | FPDM-I-3442 | Left valve |
|  |  | FPDM-I-3443 | Articulated |
|  |  | FPDM-I-3444 | Left valve |
|  |  | FPDM-I-3445 | Left valve |
|  |  | FPDM-I-3446 | Right valve |
|  |  | FPDM-I-3447 | Articulated |
| Plicatounionidae | *Plicatounio* (*Plicatounio*) *naktongensis* | FPDM-I-3448 | Butterfly position |
|  |  | FPDM-I-3449 | Left valve |
|  |  | FPDM-I-3450 | Articulated |
|  |  | FPDM-I-3451 | Articulated |
|  |  | FPDM-I-3452 | Right valve |
|  |  | FPDM-I-3453 | Left valve |
| Pseudohyriidae | *Matsumotoina* *matsumotoi* | FPDM-I-3456 | Left valve |
|  |  | FPDM-I-3457 | Right valve |
|  |  | FPDM-I-3458 | Articulated |
|  |  | FPDM-I-3459 | Articulated |
|  |  | FPDM-I-3460 | Articulated |

**Description of the color patterns in the extant freshwater bivalves from Japan**

While the color patterns of extant freshwater bivalves have been broadly illustrated, their details remain unclear. We compiled five color patterns of extant freshwater bivalves from Japan based on the specimens at Fukui Prefectural Dinosaur Museum. The terminology follows Meinhardt ^[1]^ and Caze et al. ^[2]^ and the taxonomies of Graf and Cummings ^[3]^.

Subclass: **Palaeoheterodonta** Newell, 1965 ^[4]^

Order: **Unionida** Gray, 1854 ^[5]^

Superamily: **Unionoidea** Rafinesque, 1820 ^[6]^

Family: **Unionidae** Rafinesque, 1820 ^[6]^

Subfamily: **Unioninae** Rafinesque, 1820 ^[6]^

Tribe: **Lanceolariini** Froufe, Lopes-Lima, & Bogan in Lopes-Lima et al., 2017 ^[7]^

Genus: ***Lanceolaria*** Conrad, 1853 ^[8]^

*Lanceolaria* *oxyrhyncha* (Martens, 1861) ^[9]^

Extended Data Fig. 1

*Material* *examined* —Two individuals from FPDM-I-2920, five individuals from FPDM-I-2913.

*Color* *pattern*: The color pattern consists of two components: dark green to greenish brown, 1–3 mm wide stripes along the growth lines, and the same-colored, fine radial stripes, which occasionally occur as axial segments near the ventral edge of the shells. The former is distributed mainly around the growth cessations, the latter on the middle to posterior portion of the shell. The intervals of the stripes are composed of brown to dull yellow base shell color. The color pattern of juveniles is more distinct and lighter than that of adults, with light-green to green stripes distributed on the light-brown to yellow shell.

Tribe: **Unionini** Rafinesque, 1820 ^[6]^

Genus: ***Nodularia*** Conrad, 1853 ^[8]^

*Nodularia* *douglasiae* (Griffith & Pidgeon, 1833) ^[10]^

Figure 3, Extended Data Figs. 2, 6

*Material* *examined* —Seven individuals of FPDM-I-2915, and two of FPDM-I-2917 (Extended Data Table 1).

*Color* *pattern*: The color pattern consists of three components: brown to reddish brown, 2–3 mm wide stripes along the growth cessation, green to greenish brown, 3–5 mm wide stripes distributed between each cessation, and green to greenish brown, very fine radial stripes on the whole shell surface. The intervals of the stripes are brown to dull yellow coloring, which is the base shell color. The color pattern of juveniles is more distinct and lighter than that of adults, with light-green to green stripes distributed on the light-brown to yellow shell. Two green to light-green radial stripes were distributed on the posterior portion of the shell.

Tribe: **Anodontini** Rafinesque, 1820 ^[6]^

Subtribe: **Criatariina** Lopes-Lima, Bogan & Froufe in Lopes-Lima et al., 2017 ^[7]^

Genus: ***Sinanodonta*** Modell, 1945 ^[11]^

*Sinanodonta* *calipygos* (Kobelt, 1879) ^[12]^

Figure 2, Extended Data Fig. 3

*Material* *examined* —Three individuals of FPDM-I-2924 (Extended Data Table 1).

*Color* *patterns*: The color pattern consists of two components: dark brown to dark green, 1–5 mm wide stripes along the growth lines, and dark green to green, fine radial stripes. The former is distributed mainly near the growth cessations, and the latter near the ventral edge of the shells. These color patterns vary widely among individuals, particularly the radial stripes that occasionally occur as tapered bands near the ventral edge of the shell. The intervals of the stripes are dull yellow to light-yellow coloring, which is the base shell color. The color pattern of juveniles is more distinct and lighter than that of adults, with light-green to green stripes distributed on the light-brown to yellow shell.

Genus: ***Cristaria*** Schumacher, 1817 ^[13]^

*Cristaria* *plicata* (Leach, 1814) ^[14]^

Figure 5, Extended Data Fig. 4

*Material* *examined* —Two individuals of FPDM-I-2911, and three of FPDM-I-2916 (Extended Data Table 1).

*Color* *patterns*: The color pattern consists of three components: brown to reddish brown, 2–3 mm wide stripes along the growth cessations, dark-brown to dark-green, 1–3 mm wide stripes between each cessation, and dark green to green, very fine radial stripes on the whole shell surface. These color patterns vary widely among individuals, particularly radial stripes occurring occasionally as tapered bands near the ventral edge of the shell. The intervals of the stripes are dark brown to brown, which is the base shell color. The color pattern of juveniles is more distinct and lighter than that of adults, with light-green to green stripes distributed on the light-brown to yellow shell.

Subfamily: **Gonideinae** Ortmann, 1916 ^[15]^

Tribe: **Gonideini** Ortmann, 1916 ^[15]^

Genus: ***Obovalis*** Simpson, 1900 ^[16]^

*Obovalis* *omiensis* (Heimburg, 1884) ^[17]^

Figure 4, Extended Data Figs. 5

*Material* *examined* —Two individuals of FPDM-I-2921 (Extended Data Table 1).

*Color* *patterns*: The color pattern consists of two components: brown to reddish brown, 1–3 mm wide stripes along the growth lines and dark green to greenish brown, and radial stripes, which occasionally occur as axial segments near the ventral edge of the shells. The former is distributed mainly around the growth cessations, and the latter is distributed anteriorly to the middle portion of the shell. Intervals of the stripes are brown to dull yellow coloring, which is the base shell color. The color pattern of juveniles is more distinct and lighter than that of adults, with light-green to green stripes distributed on the light-brown to yellow shell.

**Systematic Paleontology**

Subclass: **Palaeoheterodonta** Newell, 1965 ^[4]^

Order: **Trigoniida** Dall, 1889 ^[18]^

Superfamily: †**Trigonioidoidea** Cox, 1952 ^[19]^

Family: †**Trigonioididae** Cox, 1952 ^[19]^

Subfamily: †**Trigonioidinae** Cox, 1952 ^[19]^

Genus: †***Trigonioides*** Kobayashi and Suzuki, 1936 ^[20]^

*Type* *species* — †*Trigonioides* *tetoriensis* Maeda, 1963 ^[21]^

†*Trigonioides tetoriensis* Maeda, 1963 ^[21]^

Figures 1a, d, Extended Data Figs. 7–10

†*Trigonioides* *tetoriensis* Maeda, 1963 ^[21]^, pl. 12, figs. 1–9.

†*Trigonioides* *kitadaniensis* Maeda, 1963 ^[21]^, pl. 12, figs. 10–16.

†*Wakinoa tetoriensis* (Maeda, 1963) ^[21]^: Tamura (1970) ^[22]^, pl. 1, figs. 9–14, pl. 2, figs. 1, 2.

†*Trigonioides* (*Wakinoa*) *tetoriensis* Maeda, 1963 ^[21]^: Tamura (1990) ^[23]^, p. 19, pl. 2, 1–7.

†*Trigonioides* (*Wakinoa*) *tetoriensis* Maeda, 1963 ^[21]^: Matsukawa and Ido (1993) ^[24]^, p. 369, fig. 3, g.

†*Trigonioides* (*Wakinoa*) *tetoriensis* Maeda, 1963 ^[21]^: Tashiro and Okuhira (1993) ^[25]^, p. 2, fig. 2.

†*Trigonioides* (*Wakinoa*) *tetoriensis* Maeda, 1963 ^[21]^: Kozai and Ishida (2003) ^[26]^, p. 140, fig. 6, 6–7.

†*Trigonioides* *tetoriensis* Maeda, 1963 ^[21]^: Sakai et al. (2018) ^[27]^, p.177.

*Material* *examined* —Six specimens, FPDM-I-3442–3447, see Extended Data Table 2.

**Description**

*Shells* —Shell is small, equivalve, and subtrigonal in outline, with the anterior margin well rounded and posterior margin weakly curved. The shell surface is ornamented with V-shaped ribs and a strong shoulder ridge in the posterior area. Hinge teeth consist of two pseudocardinal teeth with distinct crenulations, and two posterior teeth on the left valve and one on the right valve. Anterior adductor scars are subovate in outline and deep near the pseudocardinal teeth, accompanied by a small pedal scar.

*Color* *pattern*: The color pattern consists of 7–15, 1–5 mm wide dull yellow stripes along the growth cessations on yellow ocher background with apparent 2–10 mm wide yellow ocher stripes on each interval of former stripes (Figures 1a, d, Extended Data figs 8a, c, 9a, c, 10a, c).

*Remarks* —Maeda ^[21]^ described †*Trigonioides tetoriensis* from the Lower Cretaceous Kitadani Formation, the Tetori Group in Katsuyama, Fukui. Ota ^[28]^ proposed the subgenus †*Wakinoa*, whose diagnostic features are found in †*T. tetoriensis*. Tamura ^[22]^ regards †*Trigonioides tetoriensis* as †*Wakinoa tetoriensis*, and Tamura ^[23]^ identified †*Trigonioides* (*Wakinoa*) *tetoriensis* from Kitadani Formation; however, the study does not describe the species with systematic paleontology. In this study, we identified the specimens from the Kitadani Formation as †*Trigonioides tetoriensis* based on recent taxonomy.

*Occurrence* —The Kitadani Formation, Katsuyama, Fukui, Japan. All specimens were collected from the Kitadani Dinosaur Quarry.

Family: †**Plicatounionidae** Chen, 1987 ^[29]^

Genus: †***Plicatounio*** Kobayashi and Suzuki, 1936 ^[20]^

*Type* *species* — †*Plicatounio* (*Plicatounio*) *naktongensis* Kobayashi and Suzuki, 1936 ^[20]^

†*Plicatounio* *naktongensis* Kobayashi and Suzuki, 1936 ^[20]^

Figure 1c, f, Extended Data Figs 11–15

†*Plicatounio* *naktongensis* Kobayashi and Suzuki, 1936 ^[20]^, p. 261, pl. 28, 1–4.

†*Plicatounio* *naktongensis* Kobayashi and Suzuki, 1936 ^[20]^: Suzuki (1943) ^[30]^, p. 222, pl. 16, 14–15.

†*Plicatounio* *naktongensis naktongensis* Kobayashi and Suzuki, 1936 ^[20]^: Ota (1959) ^[31]^, p. 19, pl. 3, 4–8.

†*Plicatounio* *tetoriensis* Maeda, 1962 ^[32]^, pl. 53, 5–7.

†*Plicatounio* (*Plicatounio*) *naktongensis naktongensis* Kobayashi and Suzuki, 1936 ^[20]^: Hayami (1975) ^[33]^, p. 247, pl. 9, 4–5.

†*Plicatounio* (*Plicatounio*) *naktongensis* Kobayashi and Suzuki, 1936 ^[20]^: Yang (1989) ^[34]^, p. 84, fig. 3, 3.

†*Plicatounio* (*Plicatounio*) *naktongensis naktongensis* Kobayashi and Suzuki, 1936 ^[20]^: Maeda (1990) ^[35]^, p. 11, pl. 1, 1–9, pl. 2, 1–8.

†*Plicatounio* (*Plicatounio*) *naktongensis naktongensis* Kobayashi and Suzuki, 1936 ^[20]^: Tamura (1990) ^[24]^, p. 22, pl. 3, 1–3, pl. 5, 8–10, p. 25, pl. 6, 1–3, p. 27, pl. 7, 1–2.

†*Plicatounio* (*Plicatounio*) *naktongensis* Kobayashi and Suzuki, 1936 ^[20]^: Matsukawa and Ido (1993) ^[25]^, p. 369, fig. 3, c.

*Material* *examined* —Six specimens, FPDM-I-3448–3453, see Extended Data Table 2.

**Description**

*Shells* —Shell is medium in size, equivalve, and elongated elliptical in outline, with the anterior margin rounded and posterior margin curved. The shell surface was ornamented with radial ribs and growth lines. Weak radial ribs are present on the ventral margin in the center of the shell, and five or six strong radial ribs are observed on the posterior margin. Hinge teeth consist of two pseudocardinal teeth with fine crenulations and two posterior teeth on each valve. Posterior adductor scars are trigonally ovate and larger than anterior ones. The anterior adductor scars are semicircular and deep, accompanied by a small pedal scar.

*Color* *pattern*: The color pattern consists of 5–10, 1–5 mm wide dark brown bands along the growth lines, and 2–3 mm wide dark brown axial segments arranged radially. The bands along the growth lines become clustered toward the ventral margin, and the radial segments along the intervals obscure the radial cords on the anteroventral portion of the shells. All patterns are present on the yellow ocher background color (Figures 1c, f, Extended Data fig. 12a, c, 13a, c, 14a, c, 15a).

*Remarks* —Kobayashi and Suzuki ^[20]^ proposed the genus †*Plicatounio* and described †*Plicatounio naktongensis*, from Yangpori, South Korea, and Fukuoka, Japan. Suzuki ^[30]^ described the new subspecies, †*Pl. naktongensis multiplicatus* from the Sinsyu Formation in the Rakuto series, Korea. Tamura ^[24]^ reported †*Pl.* (*Plicatounio*) *naktongensis naktongensis* and †*Pl.* (*Pl*.) *naktongensis multiplicatus* from the Kitadani Formation, without a detailed description. In this study, we identified the specimens as †*Pl*. *naktongensis* based on recent taxonomy.

*Occurrence* —The Kitadani Formation, Katsuyama, Fukui, Japan. All specimens were collected from the Kitadani Dinosaur Quarry.

Family: †**Pseudohyriidae** Kobayashi, 1968 ^[36]^

Genus: †***Matsumotoina*** Guo, 1982 ^[37]^

*Type* *species* — †*Pseudohyria matsumotoi* Yang, 1979 ^[38]^

†*Matsumotoina matsumotoi* Guo, 1982 ^[37]^

Figure 1b, e, Extended Data Figs 16–19

†*Pseudohyria matsumotoi* Yang, 1979 ^[38]^, pl. 28, 1–8.

†*Pseudohyria matsumotoi* Yang, 1979 ^[38]^: Ogasawara (1988) ^[39]^, pl. 1, 1–8, pl. 2, 1–10.

†*Pseudohyria matsumotoi* Yang, 1979 ^[38]^: Tamura (1990) ^[24]^, p. 28, pl. 8, 1–2.

†*Pseudohyria matsumotoi* Yang, 1979 ^[38]^: Tamura (1990) ^[24]^, p. 28, pl. 8, 3.

†*Pseudohyria* sp.cf. †*Ps*. *matsumotoi* Yang, 1979 ^[38]^: Tamura (1990) ^[24]^, p. 28, pl. 8, 4–15.

*Material* *examined* —Five specimens, FPDM-I-3456–3460, see Extended Data Table 2

**Description**

*Shells* —Shell is medium to large, and trigonally suboval or equivalve. The anterior margin is rounded, and the posterior margin is slightly straight. The shell surface is ornamented with radial ribs, growth lines, and several prominent growth cessations. Obscure radial ribs are present on the ventral margin in the center of the shell, whereas five or six prominent radial ribs were observed in the posterior margin. The hinge teeth are smooth, consisting of three or four pseudocardinal teeth on the left valve and two posterior teeth on both valves. The anterior adductor scars are semicircular, prominent, and accompanied by distinct pedal scars, whereas the posterior scars are subcircular and larger than the anterior adductor scars.

*Color* *pattern*: The color pattern consists of fifteen to twenty, 1–3 mm in width, black-brown bands along the growth cessations and ten to seventeen, 1–3 mm in width, dark brown radial stripes. Radial stripes along the intervals of each radial rib on the postero- to mid-ventral portion of the shell were tapered and shortened from the umbo to the ventral. All patterns are present on the yellow ocher background color (Figure 1b, e, Extended Data fig. 17a, c, 18a, c, 19a, c).

*Remarks* —MacNeil ^[40]^ proposed the genus †*Pseudohyria* and assigned †*Ps. gobiensis* as a type species. Yang ^[38]^ describes †*Ps. matsumotoi* from the Yeonhwadong Formation of the Gyeongsang Group, North Korea. Following Yang ^[38]^, Guo ^[37]^ coined the subgenus †*Matsumotoina* and assigned †*Ps. matsumotoi* Yang, 1979 ^[38]^. Fang *et al*. ^[41]^ reviewed fossil bivalves from China that were described and published from 1927 to 2007 and synonymized †*Ps*. (*Matsumotoina*) to †*Gobiella* (*Matsumotoina*). Stiller and Chen ^19^ defined the three characteristic faunas of Trigonioididean the Cretaceous, which include Jingxing fauna, *Trigonioides*-*Plicatounio* -*Nippononaia* fauna (TPN fauna), and †*Pseudohyria* fauna, and reviewed constituent genera and subgenera. Stiller and Chen ^19^ separated the †*Ps*. (*Matsumotoina*) from other †*Pseudohyria* subgenera and establishes the genus †*Matsumotoina*, which belongs to the TPN fauna. In this study, we followed Stiller and Chen ^19^ and identified the specimens as †*Matsumotoina matsumotoi*.

*Occurrence* —The Kitadani Formation, Katsuyama, Fukui, Japan. All specimens were collected from the Kitadani Dinosaur Quarry.

**References in Supplementary Information**

1. Meinhardt, H. *The algorithmic beauty of sea shells, fourth edition* (Springer-Verlag, New York, 2009).
2. Caze, B., Merle, D., Pacaud, J. M. & Saint Martin, J. P. First Systematic Study Using the Variability of the Residual Color Pattens: The Case of the Paleogene Seraphsidae (Mollusca, Gastropoda, Stromboidea). *Geodiversitas* **32**, 417–477 (2010).
3. Graf, D. L. & Cummings, K. S. Freshwater Mussels (Unionoida) of the world (and other less consequential bivalves). MUSSEL Project Web Site, http://www.mussel-project.net/. Accessed [9^th^ July] (2021).
4. Newell, N. D. Classification of the Bivalvia. *Am*. *Mus*. *Novit*. **2206**, 1–25 (1965).
5. Gray, J. E. A revision of the arrangement of the families of bivalve shells (Conchifera). *Ann*. *Mag*. *Nat*. *Hist*. **13**, 408–418 (1854).
6. Rafinesque, C. S. *Annals of nature or annual synopsis of new genera and species of animals, plants, &c. discovered in North America* (T. Smith, Lexington, Ky. 1820).
7. Lopes‐Lima, M., *et* *al*. Conservation status of freshwater mussels in Europe: state of the art and future challenges. *Biol*. *Rev*. **92**, 572–607 (2017).
8. Conrad, T. A. A synopsis of the family of Naiades of North America, with notes, and a table of some of the genera and sub-genera of the family, according to their geographical distribution, and descriptions of genera and sub-genera. *Proc*. *Acad*. *Nat*. *Sci*. *Philadelphia* **6**, 243–269 (1853).
9. Martens, E. Die japanesischen Binnenschnecken im Leidner Museum. *Malakozool*. *Blätter* **7**, 32–61 (1861).
10. Griffith, E. & Pidgeon, E. The Mollusca and Radiata. vol. 12, *in* Griffith, E. *ed*., *The animal kingdom arranged in conformity with its organization, by the Baron Cuvier, member of the Institute of France, &c. &c. &c. with supplementary additions to each order, by Edward Griffith, F.L.S.,A.S., corresponding member of the Academy of Natural Sciences of Philadelphia, &c. and others*. (London, Whittaker, 1833−1834).
11. Modell, H. Die Anodontinae, Ortm. emend. (Najad., Mollusca). Jenaische Zeitschrift für Naturwissenschaft, **78**, 58–100 (1945).
12. Kobelt, W. Fauna japonica extramarina. *Abh*. *Senckenberg*. *Nat*.*forsch*. *Ges*. **11**, 284–445 (1879).
13. Schumacher, C. F. *Essai d'un nouveau système des habitations des vers testacés* (Schultz, Copenghagen 1817).
14. Leach, W. E. *Zoological miscellany: being descriptions of new or interesting animals* (E. Nodder & Son, London 1814).
15. Ortmann, A. E. The anatomical structure of Gonidea angulata (Lea). *Nautilus* **30**, 50-53 (1916).
16. Simpson, C. T. Synopsis of the naiades, or pearly fresh-water mussels. Proc. U. S. Natl. Mus. **22**, 501–1044 (1900).
17. Heimburg, H. V. Diagnosen neuer Arten. *Nach*. *Deuts*. *Mal*. *Ges*. **16**, 92–95 (1884).
18. Dall, W. H. On the hinge of pelecypods and its development with an attempt toward a better subdivision of the group. *Am*. *J*. *Sci*. **38**, 445–461 (1889).
19. Cox, L. R. Notes on Trigoniidae, outlines the classification of the family. *Malacological Society of London,* **29**, 45–70 (1952).
20. Kobayashi, T. & Suzuki, K. Non-marine shells of the Naktong-Wakino series. *Japan. Jour. Geol. Geogr.* **13**, 243–263 (1936).
21. Maeda, S. Trigonioides from the Late Mesozoic Tetori Group, Central Japan. *Trans. Proc. Paleont. Soc. Japan. N. S*. 51, 79–85 (1963).
22. Tamura, M. The hinge structure of *Trigonioides*, with Description of *Trigonioides* *mifnensis*, sp. nov. from Upper MIfune Group, Kumamoto Pref., Japan. *Mem. Fac. Educ. Kumamoto Univ.* 18, 38–52 (1970).
23. Tamura, M. Stratigraphic and palaeontologic studies on non-marine Cretaceous bivalve faunas in southwest Japan. *Mem. Fac. Educ. Kumamoto University*, 39, 1–47 (1990) (in Japanese with English abstract).
24. Matsukawa, M. & Ido, K. Nonmarine molluscan communities and paleoecology in the Jurassic-Cretaceous Tetori Group, Japan. *Cretac. Res.* **14**, 365–381 (1993).
25. Tashiro, M. & Okuhira, K. Occurrence of *Trigonioides* from the Lower Cretaceous of Shikoku, and its significance. *Geol. Rept. Shimane Univ.* **12**, 1–9 (1993) (in Japanese with English abstract).
26. Kozai, T. & Ishida, K. Early Cretaceous non-marine bivalve faunal groups from central and eastern Shikoku, Japan. *Mem. Fukui Prefect. Dinosaur. Mus.* 133–148, (2002).
27. Sakai, Y., Sekido, S. & Matsuoka, A. Stratigraphy of Lower Cretaceous Tetori Group and stratigraphic implication of plant assemblages in border area between Ishikawa and Fukui Prefecture, central Japan. *J. Geol. Soc. Japan* **124**, 171–189 (2018) (in Japanese with English abstract).
28. Ota, Y. Notes on the relationship between *Trigonioides* and *Plicatounio*, non-marine Mesozoic Bivalvia from Eastern Asia. *Geol. Repor. Hiroshima Univ.* 12, 503–512 (1963).
29. Chen, Jin-hua. Classification and evolution of non-marine trigonioids (Bivalvia). *Advances in Science of China, Earth Sciences* **2**, 141–152 (1987). The first page of the article indicates publication in 1987, but the copyright date for the volume is marked in 1988. The 1987 date was currently accepted.
30. Suzuki, K. Restudy on the non-marine molluscan fauna of the Rakuto series in Keisyo-do, Tyosen. *J. Sigenkagaku Kenkyuuzyo* **1**, 189–219 (1943).
31. Ota, Y. Plicatounio of the Wakino Formation (studies on the molluscan fauna of the Cretaceous Inkstone series. Part 1). *Trans. Proc. Paleont. Soc. Japan. N. S*. 33, 15–18 (1959).
32. Maeda, S. Some lower Cretaceous Pelecypods from the Aikawa subgroup, the Upper division of the Tetori Group in central Japan. *Trans. Proc. Paleont. Soc. Japan. N. S*. 48, 343–351 (1962).
33. Hayami, I. A systematic survey of Mesozoic Bivalvia from Japan. *Univ. Mus., Univ. Tokyo, Bull.* 10, 1–249 (1975).
34. Yang, S. Y. On the genus Plicatounio (Cretaceous non-marine Bivalvia) from Korea. *Trans. Proc. Paleont. Soc. Japan. N. S*. 154, 77–95 (1989).
35. Maeda, S. *et* *al*. On the Plicatounio from the Hekou (Kuokou) Formation in Fujian Province, China. *Bull. Fac. Edu., Chiba Univ.* **38**, 1–13 (1990).
36. Kobayashi, T. Cretaceous non-marine pelecypods from the Nam Phung Dam site in the northeastern part of the Khorat Plateau, Thailand, with a note on the Trigonioididae. *In*: Kobayashi T, Toriyama R (eds.) *Geology and Paleontology of Southeast Asia* **4**, 109–138 (1968).
37. Guo Fu-xiang. A new subgenus Matsumotoina (Bivalvia) from the Asian non-marine Cretaceous. *Geol. Rev.* **28**, 145–147 (1982) (in Chinese with English summary).
38. Yang, S. Y. Some new Bivalve species from the Lower Gyeongsang Group, Korea. *Trans. Proc. Paleont. Soc. Japan. N. S*. 116, 223–234 (1979).
39. Ogasawara, K. Early Cretaceous freshwater mollusc from the Monomiyama Formation, Southern Kitakami Mountains, Japan. *Saito Ho-on Kai Spec. Publ.* (Professor Tamio Kotaka Commemorative Volume) 301–315 (1988).
40. MacNeil, F. S. Notes on *Pseudohyria gobiensis* gen. et sp. nov. from the Iren Dabasu Formation in Iren Dabasu, Inner Mongolia. Central Asia in Cretaceous Time. *Geol. Soc. Am. Bull.* **47** 1514–1515 (1936).
41. Fang, Zong-jie *et* *al*. Suparspecific taxa of the Bivalvia first named, described, and publishied in China (1927–2007). *Univ. Kans. paleontol. contrib.* 17, 1–157 (2009).
